# Single B Cell Gene Co-Expression Networks Implicated in Prognosis, Proliferation, and Therapeutic Responses in Non-Small Cell Lung Cancer Bulk Tumors

**DOI:** 10.3390/cancers14133123

**Published:** 2022-06-25

**Authors:** Qing Ye, Nancy Lan Guo

**Affiliations:** 1 Lane Department of Computer Science and Electrical Engineering, Statler College of Engineering and Mineral Resources, West Virginia University, Morgantown, WV 26506, USA; qiye@mix.wvu.edu; 2 West Virginia University Cancer Institute, Morgantown, WV 26506, USA; 3 Department of Occupational and Environmental Health Sciences, School of Public Health, West Virginia University, Morgantown, WV 265056, USA

**Keywords:** B cells, T cells, single-cell RNA sequencing, CRISPR-Cas9/RNAi screening, prognostic and predictive biomarkers, non-small cell lung cancer

## Abstract

**Simple Summary:**

This study presents novel insights on dysregulated B cell proliferation networks in non-small cell lung cancer (NSCLC). Within this network, a nine-gene signature demonstrated prognostic and predictive indications in more than 1400 NSCLC patients using their gene and protein expression profiles in bulk tumors. Furthermore, novel therapeutic candidates are identified to improve NSCLC treatment outcomes.

**Abstract:**

In NSCLC, there is a pressing need for immunotherapy predictive biomarkers. The processes underlying B-cell dysfunction, as well as their prognostic importance in NSCLC, are unknown. Tumor-specific B-cell gene co-expression networks were constructed by comparing the Boolean implication modeling of single-cell RNA sequencing of NSCLC tumor B cells and normal B cells. Proliferation genes were selected from the networks using in vitro CRISPR-Cas9/RNA interfering (RNAi) screening data in more than 92 human NSCLC epithelial cell lines. The prognostic and predictive evaluation was performed using public NSCLC transcriptome and proteome profiles. A B cell proliferation and prognostic gene co-expression network was present only in normal lung B cells and missing in NSCLC tumor B cells. A nine-gene signature was identified from this B cell network that provided accurate prognostic stratification using bulk NSCLC tumor transcriptome (*n* = 1313) and proteome profiles (*n* = 103). Multiple genes (*HLA-DRA*, *HLA-DRB1*, *OAS1*, and *CD74*) differentially expressed in NSCLC B cells, peripheral blood lymphocytes, and tumor T cells had concordant prognostic indications at the mRNA and protein expression levels. The selected genes were associated with drug sensitivity/resistance to 10 commonly used NSCLC therapeutic regimens. Lestaurtinib was discovered as a potential repositioning drug for treating NSCLC.

## 1. Introduction

Non-small cell lung cancer (NSCLC), accounting for 84% of lung cancer cases [1], is the leading cause of cancer-related mortality. The major histological subtypes that constitute NSCLC are lung adenocarcinoma (40% of NSCLC cases), squamous cell carcinoma (25–30%), and large cell carcinoma (5–10%), and each subtype represents a distinct prognosis for the patients as does the treatment option [2,3]. Adjuvant chemotherapy of stage II/III NSCLC has resulted in 10–15% increased overall survival [4]. However, the overall 5-year survival rate for NSCLC is less than 15% due to the limited therapeutic response and the resulted tumor recurrence/metastasis [5]. Immunotherapy has shown promising results in NSCLC [6,7]. The neoadjuvant PD-1 inhibitor nivolumab induced a pathological response in 9 of 20 (45%) resected NSCLC tumors in stages I, II, and IIIA, including both PD-1-positive and -negative tumors, with few side effects [6]. In an open-label phase I clinical trial of chemotherapy-naive NSCLC patients with stage IIIB or stage IV, nivolumab with anti-CTLA4 ipilimumab in first-line therapy had an acceptable safety profile and demonstrated promising clinical performance, with a high response rate and long-term response [7]. Patients with stage IIIB who received anti-PD-L1 durvalumab after chemoradiation had a significant overall survival benefit (median OS not reached in the durvalumab arm compared to 29.1 months in the placebo arm (HR 0.69 [95% CI 0.55–0.86])) in a phase III randomized trial [8,9]. The overall survival benefit of pembrolizumab monotherapy was demonstrated in untreated stage IV NSCLC patients compared to chemotherapy in patients with tumors expressing a PD-L1 tumor proportion score (TPS) ≥ 50%, TPS ≥ 20%, and TPS ≥ 1% in randomized phase III clinical trials [10,11,12]. Atezolizumab received FDA approval for stage II and IIIA NSCLC following chemotherapy in patients with PD-L1 > 1% [13,14]. Nevertheless, only a subset of NSCLC patients responded to immunotherapy due to primary, adaptive, or acquired immune resistance [15,16]; mutations; and the varying amounts and properties of tumor-infiltrating lymphocytes (TILs) [17,18,19]. PD-L1 and tumor mutational burdens have not been demonstrated to be reliable predictive biomarkers [20]. There are currently no well-established predictive biomarkers for immunotherapy response. In addition, there are no clinically applied biomarkers to identify early-stage NSCLC patients with all histological subtypes who are at high risk for tumor recurrence and metastasis for adjuvant therapies.

The tumor immune microenvironment is complex, dynamic, and heterogeneous, comprising interweaving signaling pathways and networks of genes and proteins within the immune system, stromal cells, and the host factors [21]. When tertiary lymphoid structures (TLSs) are present in the tumor microenvironment, cancer patients typically have favorable clinical outcomes [22]. TLSs in NSCLC tumors contain follicular B cells and adjacent clusters of dendritic cells and T cells [23]. TLSs and tumor-infiltrating B cells increase immune checkpoint inhibitors’ (ICIs) responses in cancer immunotherapy, which has prognostic implications [24,25,26,27]. Profiling of the immune cell composition showed that only B cells had a significantly higher presence in tumors compared to the distal lung in an NSCLC patient cohort [28]. A high density of B cells within a TLS is positively correlated with tumor antigen-specific antibody responses and increased intratumor CD4^+^ T cell clonality as well as early differentiated, activated, and non-regulatory CD4^+^ T cells, suggesting a central role of B cells in determining protective T cell responses in NSCLC patients [29,30]. T cell dysfunction and therapy have been established for cancer treatment, including NSCLC [31,32,33,34,35,36,37]. However, the biology, prognostic significance, and potential benefit of B-cell-based immunotherapy in lung cancer have yet to be deciphered [38,39,40].

Given this intricacy and heterogeneity, a gene signature integrating various features should be created to identify the right patient for a specific immunotherapy [21], in concert with chemotherapy and/or radiotherapy. Novel computational network modeling is necessary to reveal essential molecular interactions with implications in prognosis, proliferation, and response to therapies for improving NSCLC treatment. Rigorous pathway and network approaches are crucial for the discovery of innovative targeted therapies and repurposing drugs to prolong NSCLC survival [41]. This study utilized Boolean implication networks [42,43,44] to identify tumor-specific B cell gene co-expression networks in NSCLC using public single-cell RNA sequencing data. Within the identified B cell networks, proliferation genes were identified from CRISPR-Case9/RNA interference (RNAi) screening assays in human NSCLC cell lines. Differentially expressed genes in B cells and T cells with prognostic implications were selected using bulk NSCLC tumor transcriptome (*n* = 1313) and proteome profiles (*n* = 103). Chemopredictive genes were filtered using public in vitro drug screening data in human NSCLC epithelial cell lines (*n* = 135). Finally, functional pathways, targeted therapies, and repositioning drugs were discovered based on the genes identified using the artificial intelligence (AI) network approach for improving NSCLC outcomes.

## 2. Materials and Methods

### 2.1. Public Patient Cohorts

#### 2.1.1. Single-Cell RNA Sequencing of B Cells

A previous study [45] generated single-cell RNA sequencing data of 192 B cell samples from one NSCLC patient, including 96 tumor B cell samples and 96 normal B cell samples from adjacent normal lung tissues. Live CD45+ CD19+ cells were directly sorted in 2 µL of Qiagen TCL buffer in 96-well plates. The modified Smart-seq2 protocol [46,47] was used to prepare the libraries. The RNA samples were analyzed with Illumina HiSeq 2000. Gene features were counted with the featureCounts (version 1.4.0-p1, RRID:SCR_012919, Oxford University Press, Oxford, UK) method [48] based on the Gencode v19 transcriptome annotation. The data containing feature counts were available in the NCBI GEO database with the accession number GSE84789. Feature counts of 20,092 genes in all 192 samples were included to construct single-cell gene co-expression networks and perform differential expression analysis.

#### 2.1.2. Single-Cell RNA Sequencing of T Cells from Peripheral Blood Lymphocytes (PBLs)

Single-cell RNA sequencing data of T cells from PBLs were generated by Chiou et al. [49] at the Stanford FACS Facility. A total of 92 T cell samples were collected from three NSCLC patients, and 531 T cell samples were collected from four healthy donors. The samples were sequenced using Illumina HiSeq 4000. The reads were mapped with the *STAR* aligner [50] based on human genome reference GRCh38. SAMtools (version 1.4, RRID: SCR_002105, Oxford University Press, Oxford, UK) [51] was used to sort and index the mapped reads. The sorted PBL counts data were available in the NCBI GEO database (GSE151531). PBL counts of 17,004 genes were used in differential expression analysis in this study.

#### 2.1.3. Single-Cell RNA Sequencing of NSCLC Tumor T Cells

Tumor-infiltrating T cell data were also provided in Chiou et al. [49]. A total of 2950 T cell samples were collected from 10 NSCLC patients. The data were sequenced, mapped, sorted, and indexed with the same process as for the PBL T cell data described above. Chiou et al. [49] performed dimension reduction using Uniform Manifold Approximation and Projection (UMAP) on these NSCLC tumor T cell data and identified 14 cell clusters (C1-C14). The counts of 20,719 genes and the cell cluster identification were available in the NCBI GEO database (GSE151537).

#### 2.1.4. RNA Sequencing of Bulk NSCLC Tumors

RNA sequencing data from 199 NSCLC patient tumor samples were collected previously [52,53]. Sequencing was performed with Illumina HiSeq 2500. The reads were mapped using TopHat (version 2.0.8b, RRID:SCR_013035, Oxford University Press, Oxford, UK) [54] based on human genome reference GRCh37. The raw read count was estimated using *featureCounts* (version 1.4.0-p1, RRID:SCR_012919, Oxford University Press, Oxford, UK) [48]. Fragments per kilobase million (FPKM) data were generated with the Cufflinks tool (version 2.1.1, RRID: SCR_014597, Springer Nature, London, UK) [55]. FPKM and read counts data were available in the NCBI GEO database (GSE81089). A total of 197 samples with sufficient survival information were included for prognostic evaluation.

#### 2.1.5. Affymetrix Microarray of NSCLC Bulk Tumors

Affymetrix data of 100 NSCLC tumor samples were reported previously [56,57]. These data included samples from 53 short-term (<20 months) survivors and 47 long-term (>58 months) survivors. Transcriptional expression profiles were generated with Affymetrix Human Genome U133 Plus 2.0 Array. Raw microarray data (GSE28571) were normalized using the robust multi-array average (RMA) method. These data were used to select prognostic genes in this study.

#### 2.1.6. RNA Sequencing Data of The Cancer Genome Atlas (TCGA)

Two NSCLC cohorts from TCGA were obtained from LinkedOmics [58] (http://linkedomics.org/, accessed on 28 April 2021). These cohorts included 515 lung adenocarcinoma (LUAD) samples and 501 lung squamous cell carcinoma (LUSC) samples with matched normalized RNA sequencing data and clinical follow-up information. The TCGA datasets were used for prognostic and radiotherapy response evaluation.

#### 2.1.7. Proteomics of LUAD Tumors

Proteomic quantification of 11,056 unique genes was measured in 103 LUAD tumors using LC-MS/MS [59]. Log_10_-transformed proteomic data were used in the prognostic evaluation in this study.

### 2.2. Boolean Implication Networks

The Boolean implication network algorithm used in this study was developed by Guo et al. [42,60,61]. A detailed description of the algorithm and its applications in multi-omics network modeling was provided in our previous publications [43,44]. This algorithm was used to construct single-cell gene co-expression networks in the present study. All implication rules representing gene co-expression relations are evaluated with scope and precision determined by the algorithm. Thresholds for scope and precision were computed by a one-tailed *z*-score test with a defined significance level and sample size. In this study, we used *z* = 3.891 (α = 0.0001, one-tailed *z*-tests) for the calculation of thresholds. If an implication rule had both scope and precision values passing the threshold, that rule was kept. Among all rules that were kept for the same pair of genes, we further kept only the one with the maximum precision.

The normal and NSCLC tumor-associated B cell data were used to construct single-cell gene co-expression networks separately. In the data pre-processing for the Boolean implication network modeling, a sample with a feature count number greater than zero was denoted as “expressed”, and a sample with a feature count of zero was denoted as “not expressed” for the corresponding gene.

### 2.3. Single-Cell Differential Expression (DE) Analysis

The DE analysis of single-cell RNA sequencing data was performed with *DEsingle* [62] in Bioconductor. Genes with a *p*-value < 0.05, an absolute log_2_ fold change (FC) value > 1, and a false discovery rate (FDR) < 0.25 were considered as significantly differentially expressed.

### 2.4. Cancer Cell Line Encyclopedia (CCLE)

The CCLE provides public access to comprehensive genetic characterizations for human cancer cell lines. Data of available NSCLC epithelial cell lines were used in this study. The datasets included RNA sequencing data quantified with the GTEx pipelines [63] (*n* = 135, data obtained from https://portals.broadinstitute.org/ccle/data, accessed on 7 December 2021) and proteomic data quantified with reverse phase protein arrays [64] (*n* = 63, data obtained from https://gygi.hms.harvard.edu/publications/ccle.html, accessed on 1 October 2021).

### 2.5. CRISPR-Cas9 Knockout Assays

Whole-genome CRISPR-Cas9 knockout screening results in CCLE cell lines were generated in Project Achilles [63]. The genome-wide dependency scores in 94 NSCLC cell lines were obtained from the DepMap portal version 21Q4 data release [65] (https://depmap.org/portal/download/, accessed on 7 December 2021). A normalized dependency score less than −0.5 indicates a significant effect of CRISPR-Cas9 knockout.

### 2.6. RNAi Knockdown Assays

Whole-genome RNAi screening data for CCLE cell lines were also generated in Project Achilles [63] (https://depmap.org/R2-D2/, accessed on 1 April 2021). The genome-wide dependency scores in 92 NSCLC cell lines were used for analysis in this study. RNAi knockdown was considered to have a significant effect if the normalized dependency score was smaller than −0.5.

### 2.7. PRISM Drug Response in CCLE

The PRISM dataset [66] was obtained from the DepMap portal (https://depmap.org/portal/download/, PRISM repurposing 19Q4, accessed on 1 April 2021). Carboplatin, cisplatin, docetaxel, erlotinib, etoposide, gefitinib, gemcitabine, paclitaxel (Taxol), pemetrexed, and vinorelbine are commonly used chemotherapeutic drugs in NSCLC treatment, so our analysis in this study was focused on these ten regiments. Drug activity measurements, including IC_50_, ln(IC_50_), EC_50_, and ln(EC_50_), were used in the analysis. The available CCLE NSCLC cell lines were divided into three groups according to the drug activity measurements: sensitive, resistant, and intermediate, as described previously.

### 2.8. Genomics of Drug Sensitivity in Cancer (GDSC)

Drug activity measurements were generated in the GDSC Project [67] (https://www.cancerrxgene.org/downloads/bulk_download, accessed on 15 April 2021). IC_50_ and ln(IC_50_) were the main metrics used in this analysis. The available CCLE NSCLC cell lines were divided into three groups based on the drug activity measurements in the GDSC1/2 datasets, namely sensitive, resistant, and intermediate, as described previously [43,44].

### 2.9. Drug Repurposing Using Connectivity Map (CMap)

CMap (RRID:SCR_016204, Cell Press, Cambridge, MA, USA) [68,69] provides a tool to identify genes, drugs, and disease states connected with gene expression signatures. By matching input gene expression signatures, candidate functional pathways and repositioning drugs can be generated with CMap. The results with a *p*-value < 0.05 and a connectivity score > 0.9 can be used as hypotheses for further investigation. The upregulated and downregulated gene lists discovered in this study were used as database queries to find the connected functional pathways and reposition drugs.

### 2.10. Pathway Enrichment Analysis Using ToppGene

The ToppGene [70] Suite (RRID:SCR_005726, https://toppgene.cchmc.org, accessed on 8 March 2022) provides several tools for interpreting gene functional classification and enrichment analysis. The ToppFun tool of the ToppGene suite was used to find the statistical enrichment of pathways in this study.

### 2.11. Statistical Methods

RStudio (version 1.4.1106) [71], an integrated development environment for R, was used as the main tool for performing the statistical analyses. A two-tailed, unpaired Student’s *t*-test was used to evaluate the significance of differential expression between two groups. Survival analysis and curves were generated using the Kaplan–Meier method, and log-rank tests were used to assess the difference in survival probability between different groups. Univariate and multivariate Cox regression analyses were used in the prognostic evaluation and curve generation with R packages “*survival* (version 3.3.1, Springer, New York City, NY, USA)”, “survminer (version 0.4.9, https://cran.r-project.org/package=survminer)”, and “ggplot2 (version 3.3.6, Springer-Verlag, New York City, NY, USA)”. The strength of the linear relationship between two sample groups was measured with Pearson’s correlation test. Results in statistical tests were considered significant with a *p*-value < 0.05.

## 3. Results

### 3.1. Tumor-Specific Gene Co-Expression Networks in NSCLC B Cells

Using the Boolean implication network algorithm, whole-genome mRNA co-expression networks were generated with single-cell RNA sequencing data of normal and NSCLC tumor B cells. The constructed tumor and normal B cell gene co-expression networks were compared to identify tumor-specific B cell co-expression networks, i.e., gene co-expression relations that existed only in tumor B cells and not in normal B cells, and vice versa. To obtain a manageable amount of network edges, we selected the significant (*p* < 0.00005, one-tailed *z*-tests) gene associations (i.e., implication rules) for further analysis. A total of 232 significant gene co-expression relations (network edges) existed only in the NSCLC tumor B cell network and not in the normal B cell network, and 615,332 significant co-expression relations existed only in the normal B cell network and not in the NSCLC tumor B cell network. These identified tumor-specific B cell gene co-expression networks were included in further analysis.

### 3.2. DE Genes in NSCLC B Cells and T Cells with Prognostic Implications

We included the significant DE genes (*p* < 0.05, |log_2_FC| > 1, and FDR < 0.25) in tumor vs. normal B cells in the next analysis. A total of 1086 unique genes were selected, including 1082 genes from the network only present in normal B cells and 35 genes from the network only present in NSCLC tumor B cells.

**Figure 1:**
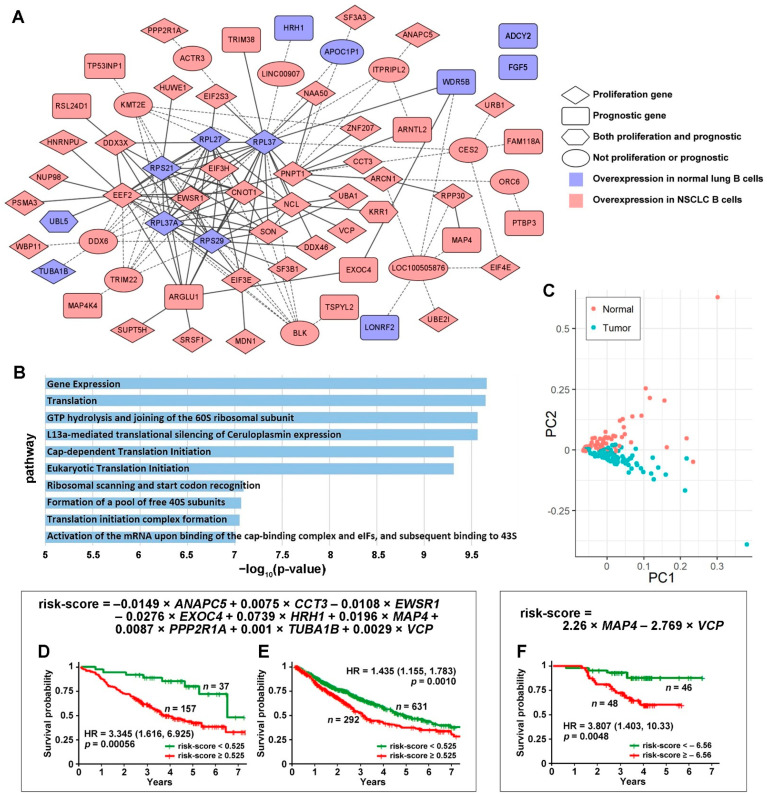
The identified proliferation and prognostic gene co-expression network in NSCLC B cells. (**A**) The shown gene co-expression network was present in normal B cells and missing in NSCLC tumor B cells. None of the co-expression relations were present in tumor B cells. All genes were significantly differentially expressed in NSCLC tumor-associated B cells vs. B cells in adjacent normal lung tissues. The intermediate genes are in the ellipse circles. These genes were not in the selected proliferation or prognostic gene list, but the selected genes were connected through these intermediate genes. The solid lines indicate direct connections between the selected genes, and the dashed lines indicate connections through intermediate genes. (**B**) The −log_10_ (*p*-value) of the top 10 significantly enriched pathways in the ToppGene functional enrichment analysis of the proliferation and prognostic network. (**C**) Principal component analysis (PCA) using all the genes shown in (**A**) in single-cell RNA sequencing data separates normal and NSCLC tumor B cells. Kaplan–Meier analysis of the 9-gene signature using RNA sequencing data in the training set GSE81089 (**D**) and the TCGA-LUAD and TCGA-LUSC validation set (**E**). (**F**) Kaplan–Meier analysis of the 9-gene signature using proteomic data of MAP4 and VCP in LUAD patients.

Proliferation and prognostic genes were further selected from these 1086 DE genes (Supplementary File S1, Tables S1 and S2). The proliferation genes that had a significant effect in both CRISPR-Cas9 and RNAi screening assays in more than 50% of the tested human NSCLC epithelial cell lines were selected (Figure 1A, Supplementary File S2). The prognostic genes that had a significant hazard ratio (HR, *p* < 0.05, univariate Cox model) in the survival analysis of an NSCLC cohort (GSE81089) and significant differential expression (*p* < 0.05, two-sample *t*-tests) between short- and long-term NSCLC survivors were selected (GSE28571). The selected prognostic genes had a concordant association with patient survival in both cohorts. Detailed information on the selected prognostic genes is provided in Table S1.

The identified proliferation and prognostic gene co-expression network (Figure 1A) was only present in the normal B cells and was missing in the NSCLC tumor B cells. Some genes were not directly connected with the network but were connected through one of the intermediate genes differentially expressed in NSCLC tumor B cells. *ADCY2* and *FGF5* were not connected with the network directly or through any intermediate genes. Detailed information on the network edges is provided in Table S2. The B cell proliferation and prognostic network pathway analyses were conducted with ToppGene. The top 10 significantly enriched pathways are shown in Figure 1B. Detailed information of all significantly (*p* < 0.05, FDR B&H and FDR B&Y < 0.25) enriched pathways of the B cell proliferation and prognostic network (Figure 1A) is provided in Table S3. The principal component analysis (PCA) using all the genes shown in Figure 1A generated a clear separation of normal and NSCLC tumor B cells in single-cell RNA sequencing data (Figure 1C).

From the B cell proliferation and prognostic gene co-expression network (Figure 1A), a nine-gene prognostic signature was identified in the multivariate Cox model from the training set (GSE81089) and was validated in the combined TCGA-LUAD and TCGA-LUSC data of NSCLC patients with stage I, II, or IIIA (details provided in Supplementary File S3). In the multivariate Cox model analysis, a stepwise gene selection method was used to construct the model in the training set. In each iteration, the least statistically significant gene variable in the multivariate Cox model was dropped. This iteration was repeated until the model with the optimal prognostication in the training set was achieved. This model was then validated on the test set (TCGA data) with all the training parameters fixed. Nine marker genes were selected in this process: *ANAPC5*, *CCT3*, *EWSR1*, *EXOC4*, *HRH1*, *MAP4*, *PPP2R1A*, *TUBA1B*, and *VCP*. The Kaplan–Meier analysis results showed that the patients with a risk score lower than 0.525 survived significantly longer than the patients with a risk score higher than 0.525 in both the training (*p* = 0.00056; HR: 3.345 [1.616, 6.925]; Figure 1D) and validation cohorts (*p* = 0.0010; HR: 1.435 [1.155, 1.783]; Figure 1E). The detailed TCGA data used in the validation are provided in Supplementary File S3.

The protein expression of the nine-gene marker panel also provided significant patient stratification in a LUAD patient cohort [59]. Only MAP4 and VCP were included in the proteomic multivariate Cox model due to data availability and the univariate significance. The Kaplan–Meier analysis results showed that the patient group with a risk score lower than −6.56 survived significantly longer than the patient group with a risk score higher than −6.56 (*p* = 0.0048; HR: 3.807 [1.403, 10.33]; Figure 1F).

**Figure 2:**
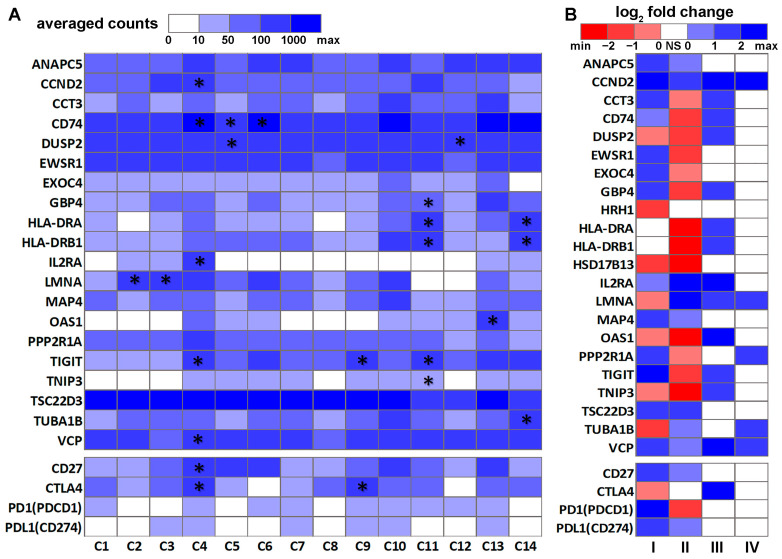
Differential expression patterns of the selected genes in NSCLC T cells and B cells. (**A**) Heatmap of the average expression of the selected genes in 14 NSCLC tumor T cell clusters [49]. The asterisk (*) indicates that the gene was significantly differentially expressed in the corresponding T cell cluster [49]. (**B**) Heatmap of the log_2_FC patterns of the selected genes in this study. I: NSCLC tumor B cells (*n* = 96) vs. normal B cells (*n* = 96) [45]. II: Peripheral blood lymphocyte T cells from NSCLC patients (*n* = 531) vs. healthy donors (*n* = 92) [49]. III: NSCLC suppressive tumor Tregs of CD4-C9-CTLA4 cells (*n* = 868) vs. other tumor-infiltrating Tregs of CD4-C8-FOXP3 cells (*n* = 122) [73]. IV: NSCLC activated tumor Tregs of CD4-C9-CTLA4 (TNFRSF9^+^, *n* = 519) vs. non-activated tumor Tregs of CD4-C9-CTLA4 (TNFRSF9^−^, *n* = 420) [73]. NS: not significant.

Interactions and coordination between B cells and T cells are crucial in antibody generation and immune protection [72]. To gain a better understanding of genes important in NSCLC immunogenetics, we next examined DE genes in T cells in NSCLC PBL and tumors. Firstly, significant DE genes (*p* < 0.05, |log_2_FC| > 1, and FDR < 0.25) between normal and NSCLC PBL T cells (GSE151531) were identified. Secondly, this list of DE genes was compared with two T cell DE gene lists from published studies [49,73]. One published gene list [73] contained DE genes (*p* < 0.05, two-sided moderated *t*-tests) between suppressive tumor Tregs of CD4-C9-CTLA4 cells (*n* = 868) vs. other tumor-infiltrating Tregs of CD4-C8-FOXP3 cells (*n* = 122) as well as DE genes (*p* < 0.05, two-sided moderated *t*-tests) between activated tumor Tregs of CD4-C9-CTLA4 (TNFRSF9^+^, *n* = 519) vs. non-activated tumor Tregs of CD4-C9-CTLA4 (TNFRSF9^−^, *n* = 420). The other published gene list [49] consisted of the DE genes in each of the 14 T cell clusters. Eleven genes, including *CCND2*, *CD74*, *DUSP2*, *GBP4*, *HLA-DRA*, *HLA-DRB1*, *IL2RA*, *LMNA*, *OAS1*, *TIGIT*, and *TNIP3*, were common DE genes in all three lists. Two genes, *HSD17B13* and *TSC22D3*, had concordant significant DE (*p* < 0.05, |log_2_FC| > 1, and FDR < 0.25) between NSCLC vs. normal PBL T cells (GSE151531) and B cells in the lung tissues (GSE84789). These selected 22 genes, including 13 DE genes and the nine-gene prognostic marker panel identified from the B-cell network (Figure 1A), and 4 ICIs (*CD27*, *CTLA4*, *PD1*, and *PDL1*) were visualized in heatmaps (Figure 2). CTLA4, PD1, and PD-L1 are well-established immunotherapy targets in NSCLC [74]. CD27 is a new ICI [75] that is being investigated in phase I/II clinical studies for a variety of cancer types, with encouraging results [76,77]. CD27 was also included in a seven-gene prognostic and chemopredictive assay we identified previously, showing concordant prognostic indications at the mRNA and protein expression levels in NSCLC tumors [44,78]. Here, we sought to examine the expression of these ICIs in NSCLC tumor B cells, T cells, and PBL T cells in single-cell RNA sequencing profiles. The average expression of these genes in the 14 NSCLC T cell clusters [49] is shown in Figure 2A, and their DE patterns in NSCLC T cells and B cells are shown in Figure 2B. *HRH1* and *HSD17B13* were not available in the NSCLC tumor T cell dataset [49] and are therefore not included in Figure 2A.

The 14 T cell clusters presented in Figure 2A were published by Chiou et al. [49]. In this analysis of the Stanford cohort, 14 major distinct T cell states of activation/exhaustion were identified [49], of which 13 (C1 to C13) could be linked to the cell states reported by Guo et al. [73] using a different patient cohort. Cluster C14 from Chiou et al. [49] represented CD4^+^ and CD8^+^ T cells in the cell cycle, which was not reported by Guo et al. [73]. Clusters C5, C6, and C12 were CD8^+^ T cells with effector phenotypes. Clusters C7 and C10 were CD8^+^ T cells with a resident memory phenotype. C11 [49]/CD8-C6-LAYN [73] (CD8^+^ exhausted T cells) and C4 [49]/CD4-C9-CTLA4 [73] (CD4^+^ Tregs) consisted almost entirely of cells originated from tumors. Clusters C5, C6, C7, and C12 were inferred to exhibit virus-specific T cell states [49].

**Figure 3:**
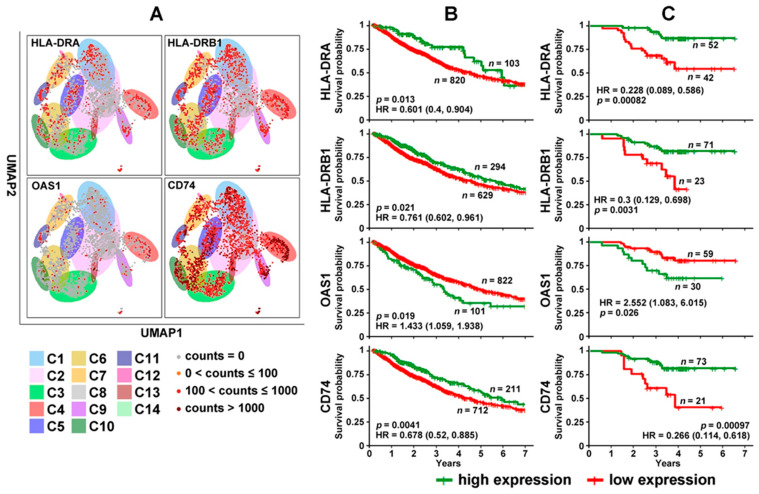
Differentially expressed genes in NSCLC T cells with prognostic indications. (**A**) Expression of *HLA-DRA*, *HLA-DRB1*, *OAS1*, and *CD74* in 2950 single T cells across 14 clusters illustrated in the UMAP layout. (**B**) Kaplan–Meier analysis of TCGA-LUAD and TCGA-LUSC patients stratified based on mRNA expression of *HLA-DRA*, *HLA-DRB1*, *OAS1*, and *CD74* in the RNA-sequencing data. (**C**) Kaplan–Meier analysis of patients in the Xu cohort [59] stratified based on the protein expression of HLA-DRA, HLA-DRB1, OAS1, and CD74.

Among the selected genes (Figure 2), HLA-DRA, HLA-DRB1, OAS1, and CD74 had concordant prognostic indications at the mRNA and protein expression levels in bulk NSCLC tumors with stage I, II, or IIIA (Figure 3). Figure 3A presents their mRNA expression in 2950 single NSCLC T cells across 14 clusters (GSE151537) illustrated in the UMAP layout. *HLA-DRA* and *HLA-DRB1* were both DE genes in C11 [49]/CD8-C6-LAYN [73] (CD8^+^ exhausted T cells) and the C14 cluster of CD4^+^ and CD8^+^ cells in the cell cycle [49] (Figure 2A). *OAS1* was a DE gene in C13 [49]/C4-GZMK and C4-CD69 [73] CD4^+^ cells. *CD74* was a DE gene in C4 [49]/C9-CTLA4 [73] CD4^+^ cells and the C5 and C6 clusters of CD8^+^ effector T cells. The results showed that the patients with a higher expression of HLA-DRA, HLA-DRB1, and CD74 survived significantly longer (*p* < 0.05, Kaplan–Meier analysis) than those with a lower expression of these genes at both the mRNA (Figure 3B) and protein levels [59] (Figure 3C). When OAS1 was expressed more highly at both the mRNA and protein levels, patients survived for a significantly shorter duration (*p* < 0.05, Kaplan–Meier analysis) in both TCGA-LUAD and TCGA-LUSC cohorts (Figure 3B) and the proteomic LUAD cohort from Xu et al. [59] (Figure 3C). Detailed expression cut-off values are provided in Table S4. Although different patient cohorts were used in the single-cell and prognostic analysis, these results indicate the potential utility of the identified genes in single B cell and T cell analysis of NSCLC patient tumors and PBLs for diagnosis and prognosis.

### 3.3. Genes Associated with Response to Radiotherapy and Chemotherapy

**Figure 4:**
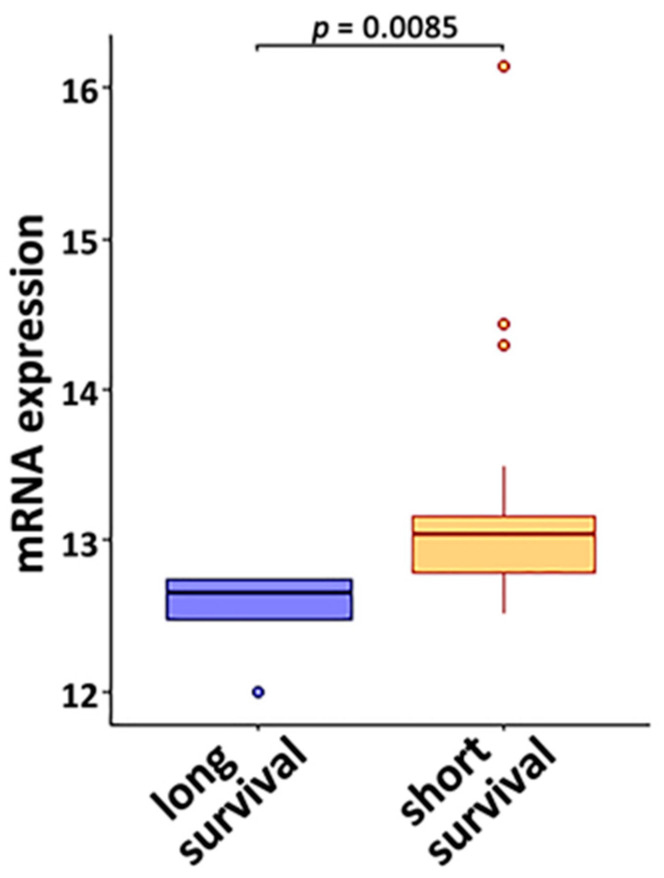
The mRNA expression of *VCP* was associated with resistance to radiotherapy. The studied patient cohort was TCGA-LUSC and TCGA-LUAD stage III and stage IV patients who had been treated with radiotherapy. *VCP* showed a significantly higher expression level (*p* = 0.0085, two-sample student *t*-tests) in the short-survival patient group (<20 months; *n* = 186) compared with the long-survival patient group (>58 months; *n* = 144). The dots were outliers that were out of the interval [Q1 − 1.5 × IQR; Q3 + 1.5 × IQR] (Q1: quartile 1, refers to 25th percentile; Q3: quartile 3, refers to 75th percentile; IQR = interquartile range from Q1 to Q3).

To assess the association with radiotherapy response, the mRNA expression of the selected genes (Figure 2) was evaluated in the TCGA-LUAD and TCGA-LUSC cohorts. The cohorts only included the stage III and stage IV patients who received radiotherapy (*n* = 56). *VCP* had a significantly higher mRNA expression level (*p* < 0.05, two-sample Student’s *t*-tests) in the short-survival (<20 months) patient group than in the long-survival (>58 months) patient group (Figure 4). The averaged log_2_-transformed RPKM of VCP was 12.52 in the long-survival patient group and 13.22 in the short-survival patient group. The average original RPKM of VCP was 5975.11 in the long-survival patient group and 12367.55 in the short-survival patient group. The fold change of VCP was 2.07 in the short-survival vs. the long-survival patient group who received radiotherapy using the original gene expression measurements.

We next examined the association of the 22 selected DE genes (Figure 2) with the drug sensitivity profiles of selected NSCLC chemotherapeutic drugs in 135 NSCLC cell lines. Lists of the genes that were significantly differentially expressed (*p* < 0.05; two-sample *t*-tests) in sensitive NSCLC cell lines vs. resistant NSCLC cell lines at the mRNA level (Table 1) and protein level (Table 2) are provided.

### 3.4. Discovery of Targeted Therapies and Repositioning Drugs

We previously developed a seven-gene prognostic and predictive assay for early-stage NSCLC (including *ABCC4*, *CCL19*, *SLC39A8*, *CD27*, *FUT7*, *DAG1*, and *ZNF71*) [44,78]. ZNF71 protein and its KRAB isoform gene expression are associated with epithelial to mesenchymal transition (EMT) [44,79]. We also reported a 14-gene classifier characterizing NSCLC tumor EMT states that were associated with distinct patient survival outcomes [79]. To improve NSCLC treatment outcomes, targeted therapies and repositioning drugs were searched in the CMap database [68,69] to prolong survival, enhance drug response, inhibit NSCLC proliferation, and reverse EMT. The CMap input gene list included the nine-gene prognostic marker panel identified from the B cell network (Figure 1A), drug-sensitive and -resistant genes (Table 1), six proliferation genes with a significant effect in both CRISPR-Cas9 and RNAi assays of all the tested human NSCLC cell lines, and EMT genes used in our previous CMap analysis [44]. Drug-sensitive/resistant genes with conflicting results between mRNA (Table 1) and protein expression (Table 2) were removed. One additional gene, *CDK7*, was added to the CMap input gene list. *CDK7* was associated with *DAG1* in our previously developed seven-gene NSCLC prognostic and predictive marker panel [78] in the NSCLC B cell networks. Specifically, a co-upregulation of *CDK7* and *DAG1* was observed in normal lung B cells, but not in the tumor B cells (*p* < 0.05, one-tailed *z*-tests). *CDK7* was significantly overexpressed (log_2_FC = 2.92, *p* < 0.05, and FDR < 0.25) in NSCLC tumor B cells vs. normal B cells. *CDK7* was a survival hazard gene with an HR greater than one (*p* < 0.05, univariate Cox model) in GSE81089 and a fold change (short-survival/long-survival) greater than one (*p* < 0.05, two-sample *t*-tests) in GSE28571. *CDK7* had a significant knockout effect in CRISPR-Cas9 assays in all 94 tested human NSCLC cell lines. The mRNA expression of *CDK7* was associated with drug resistance to cisplatin and erlotinib in human NSCLC cell lines (*n* = 135).

**Figure 5:**
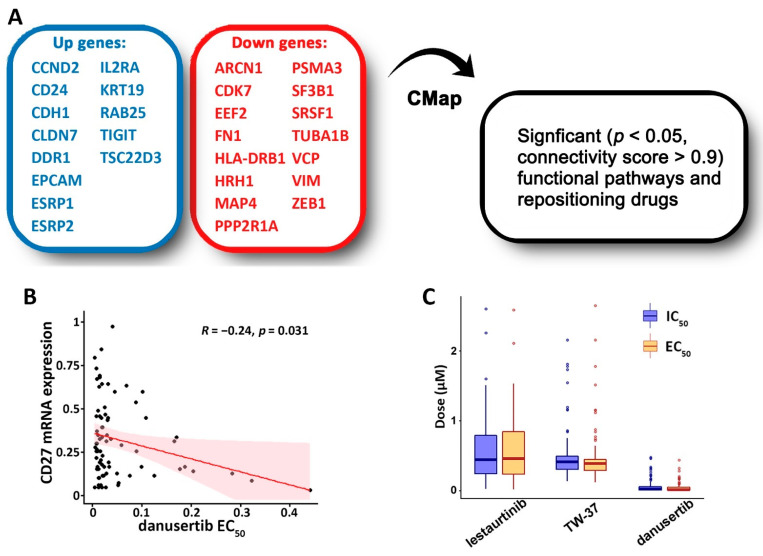
Discovery of repositioning drugs and functional pathways based on the selected genes. (**A**) Selection of significant functional pathways and repositioning of drugs based on the identified B cell proliferation and prognostic network with CMap. (**B**) The Pearson correlation of *CD27* mRNA expression and danusertib EC_50_ in NSCLC cell lines (*n* = 79). (**C**) CMap selected compounds that had a low average concentration of drug response (IC_50_ and EC_50_) in the CCLE NSCLC cell lines (n_lestaurtinib_ = 67, n_TW-37_ = 81, n_danusertib_ = 80).

The following mechanisms were applied to identify functional pathways, targeted therapies, and repositioning drugs for improving NSCLC treatment: (1) upregulation of survival-protective genes; (2) downregulation of survival hazard genes; (3) upregulation of drug-sensitive genes; (4) downregulation of drug-resistant genes; (5) downregulation of proliferation genes; (6) upregulation of the epithelial markers selected from our previous work [44,79]; (7) downregulation of mesenchymal markers that we previously reported [44,79]. The input upregulated and downregulated gene lists (Figure 5A, Supplementary Table S5) for CMap analysis were created based on these mechanisms, and genes present in both up- and downregulated lists were removed.

Several targeted therapeutic candidates were identified with CMap (Table S6). First, compounds were selected as targeted therapies that had a significant negative correlation (*p* < 0.05, Pearson’s correlation test) between drug response measurements (IC_50_, ln(IC_50_), EC_50_, ln(EC_50_)) and gene expression of one of four ICIs (*CD27*, *PD1*, *CTLA4*, or *PDL1*) in human NSCLC epithelial cell lines (*n* = 135). The EC_50_ of danusertib had a significant negative correlation (*p* < 0.05, Pearson’s correlation test) with *CD27* mRNA expression (Figure 5B). Second, drugs were selected if they had a low average IC_50_ and EC_50_ in human NSCLC epithelial cell lines (*n* = 135), indicating their efficacy in inhibiting NSCLC cell growth. Three small molecules were selected, namely danusertib, lestaurtinib, and TW-37 (Figure 5C), which had low average IC_50_ and EC_50_ values (less than 1 μM) without any outliers (IC_50_ or EC_50_ higher than 10 μM) in NSCLC cells.

Lestaurtinib is a tyrosine kinase inhibitor of fms-like tyrosine kinase 3 (FLT3) for treating AML [80,81]. The results from this study suggest that lestaurtinib could be repositioned to improve NSCLC treatment when combined with existing therapies. Evidence supports the use of the other selected small molecules for NSCLC treatment. Danusertib, an aurora kinase inhibitor [82], was investigated in treating advanced solid tumors including NSCLC in phase I [83] and phase II [84] clinical studies, in which single-agent danusertib was well tolerated and showed marginal anti-tumor activity in common solid tumor types. TW-37, a small molecule inhibitor of B-cell lymphoma 2 (Bcl-2) family proteins [85], enhanced the pro-apoptosis and anti-migration ability of gefitinib in NSCLC [86]. TG-101348, an inhibitor of Janus kinase 2 (JAK2), reduced PD-L1 protein expression [87] and reversed erlotinib resistance in NSCLC cells [88]. TG-101348 mediated radiosensitization and EMT blockade in a xenograft mouse model of lung cancer [89]. These results in the literature support the effectiveness of the presented artificial intelligence pipeline for identifying targeted therapies and repositioning drugs. 

Significant functional pathways (*p* < 0.05, connectivity score > 0.9) in overexpression and shRNA knockdown assays of NSCLC cell lines were identified using CMap that matched the input gene expression signature (Table 3). The identified significant functional pathways included metabolism; VEGF; DNA synthesis and repair; protein regulation; ribosomal subunit (40S), which is important in the scanning of mRNAs and initiation of protein synthesis [90]; and hallmarks, including PI3K, STAT3, and BRAF, etc., in shRNA knockdown assays. Overexpression of cell cycle inhibition was also identified as a significant functional pathway (Table 3). It should be noted that although the results from various functional assays were used to select these significant pathways via bioinformatics matching, they can only be interpreted as hypotheses for future investigations.

## 4. Discussion

This study identified a gene co-expression network observed only in normal B cells (Figure 1A) and missing in NSCLC tumor B cells. The transcriptomic and proteomic profiles of the nine-gene prognosis marker panel identified from this B cell network accurately stratified resectable NSCLC patients into different groups with distinct survival outcomes. A similar approach was applied to dissect tumor-specific B cell proliferation networks involving *PD1* (*PDCD1*), *PDL1* (*CD274*), and *CTLA4* (Figure S1 and Tables S7 and S8). Each gene associated with these three established ICIs (Figure S1) was significantly differentially expressed in NSCLC tumor B cells vs. normal B cells and had a significant impact on proliferation in CRISPR-Cas9 and/or RNAi assays in more than 50% of the tested human NSCLC cell lines. *VCP*, also present in Figure 1A, was co-upregulated with *PDL1* (*p* < 0.05, one-tailed *z*-test). *VCP* was overexpressed in NSCLC tumor B cells, NSCLC PBLs, and tumor T cells (Figure 2), as well as a C4 [49]/C9-CTLA [73] CD4 T cell cluster. *VCP* was in the nine-gene prognostic marker panel as a survival hazard gene and was associated with radiotherapy resistance (Figure 4) in NSCLC patients. Consistently, VCP protein expression was correlated with metastasis and poor prognosis in NSCLC patients [91]. VCP mRNA and/or protein expression was associated with drug sensitivity to cisplatin, etoposide, paclitaxel, vinorelbine, and gemcitabine and resistance to gefitinib (Table 1 and Table 2).

Evidence supports the therapeutic significance of the other genes in the nine-gene prognostic marker panel identified from the B cell network (Figure 1A). *ANAPC5* was overexpressed in NSCLC tumor B cells and PBL T cells (Figure 2) and was associated with resistance to cisplatin in NSCLC epithelial cells (Table 1). ANAPC5 protein expression was associated with drug sensitivity to pemetrexed (Table 2). Depletion of *ANAPC5* exhibited a synthetic lethal interaction with paclitaxel in NSCLC cells, suggesting enhanced sensitivity to *APC/C* inhibition in the tumor cells [92]. *CCT3*, a survival hazard gene identified in this study, was overexpressed in NSCLC tumor B cells and suppressive tumor Tregs of CD4-C9-CTLA cells vs. other tumor infiltrating Tregs of CD4-C8-FOXP3 cells [73]. *CCT3* was underexpressed in NSCLC PBL T cells (Figure 2). Suppression of *CCT3* inhibited tumor progression through the impairment of ATP production and cytoplasmic translation in lung adenocarcinoma [93]. CCT3 mRNA/protein expression was associated with drug sensitivity to erlotinib, etoposide, gefitinib, gemcitabine, pemetrexed, and vinorelbine (Table 1 and Table 2). *HRH1*, an NSCLC survival hazard gene found in this study, was underexpressed in NSCLC tumor B cells and was associated with resistance to erlotinib and vinorelbine in human NSCLC epithelial cell lines. HRH1 protein expression was associated with sensitivity to pemetrexed (Table 2). *HRH1* was reported as an apatinib (VEGFR2 inhibitor) upregulated gene and was also associated with neuroactive ligand in NSCLC cells [94]. *HRH1* had different prognostic implications in various cancer types [95]. *TUBA1B*, an identified survival hazard gene, was underexpressed in NSCLC tumor B cells. *TUBA1B* was overexpressed in NSCLC PBL T cells, C14 CD4/CD8 T cell clusters [49], and activated tumor Tregs of CD4-C9-CTLA4 (TNFRSF9^+^) vs. non-activated tumor Tregs of CD4-C9-CTLA4 (TNFRSF9^−^) [73]. TUBA1B mRNA and/or protein expression was associated with resistance to gefitinib, docetaxel, and paclitaxel and sensitivity to etoposide (Table 1 and Table 2). TUBA1B protein expression differed significantly between NSCLC patients and healthy individuals and the difference was correlated with the lipid response [95]. These results indicate that the identified network missing in NSCLC tumor B cells (Figure 1A) has important implications for proliferation, prognosis, and therapeutic responses. The pathway enrichment analysis of this network (Figure 1B) showed over-representation in ribosomal scanning, protein synthesis, and metabolism, consistent with the results from the functional assays of NSCLC cell lines matching the gene expression signature associated with clinical phenotypes (Table 3). These results shed light on dysregulated gene expression networks in singular B cells with prognostic and therapeutic significance in NSCLC.

Among the identified DE genes in NSCLC PBL T cells and tumor T cell clusters [49,73], HLA-DRA, HLA-DRB1, OAS1, and CD74 had concordant prognostic indications at the mRNA and protein expression levels in bulk NSCLC tumors with resectable disease. *HLA-DRA* and *HLA-DRB1* belong to HLA class II alpha and beta chain paralogs, respectively. Both *HLA-DRA* and *HLA-DRB1* were underexpressed in NSCLC PBL T cells and overexpressed in NSCLC tumor C11 [49]/C6-LAYN [73] CD8 T cell clusters, C14 CD4/CD8 T-cell clusters [49], and suppressive tumor Tregs of CD4-C9-CTLA4 cells vs. other tumor-infiltrating Tregs of CD4-C8-FOXP3 cells [73]. A higher mRNA and protein expression of HLA-DRA and HLA-DRB1 in bulk NSCLC tumors was associated with prolonged patient survival (Figure 3). HLA-DRA protein expression was associated with sensitivity to gefitinib and resistance to paclitaxel (Table 2). *HLA-DRB1* mRNA expression was associated with resistance to carboplatin (Table 1). HLA-DR protein expression was decreased in tumor-infiltrating immune cells and regional lymph nodes of NSCLC [96]. *OAS1* was overexpressed in C13 CD4 T cell clusters [49]/C4-GZMK/C4-CD69 CD8/CD4 T cell clusters [73] and suppressive tumor Tregs of CD4-C9CTLA cells vs. other tumor-infiltrating Tregs of CD4-C8-FOXP3 cells [73]. *OAS1* was underexpressed in NSCLC tumor B cells and PBL T cells (Figure 2). OAS1 mRNA and protein expression was associated with poor prognosis in NSCLC patients (Figure 3). OAS1 mRNA/protein expression was associated with resistance to cisplatin and gefitinib and sensitivity to paclitaxel (Table 1 and Table 2). Increased *OAS1* expression in cancer cells promotes their ability to survive DNA damage by attenuating Poly(ADP-ribose) synthesis and thus preventing cell death [97]. *CD74* was overexpressed in C4 [49]/C9-CTLA4 [73] CD4 T cell clusters, C5 and C6 [49]/C4-GZMK [73] CD8 T cell clusters, NSCLC tumor B cells, and suppressive tumor Tregs of CD4-C9-CTLA4 cells vs. other tumor-infiltrating Tregs of CD4-C8-FOXP3 cells [73]. *CD74* was underexpressed in NSCLC PBL T cells (Figure 2). Higher CD74 mRNA and protein expression was associated with a good prognosis in NSCLC patients (Figure 3). CD74 protein expression was associated with sensitivity to gemcitabine (Table 2). *CD74-NRG1* fusions, originally found in non-smoking lung adenocarcinoma patients, provide the ligand for ERBB2-ERBB3 receptor complexes through the extracellular expression of the EGF-like domain of NRG1 III-β3 [98]. *CD74-NTRK1* fusions are oncogenic and lead to constitutive TRKA kinase activity in lung cancer [99].

*TIGIT* was overexpressed in C4 [49]/C9-CTLA [73] CD4 T cell clusters, C5 and C6 [49]/C4-GZMK [73] CD8 T cell clusters, NSCLC tumor B cells, and suppressive tumor Tregs of CD4-C9CTLA cells vs. other tumor-infiltrating Tregs of CD4-C8-FOXP3 cells [73]. *TIGIT* was underexpressed in NSCLC PBL T cells (Figure 2). *TIGIT* gene expression was associated with sensitivity to carboplatin and gemcitabine (Table 1). The immunomodulatory receptor *TIGIT* is an emerging ICI for cancer immunotherapy [100]. Tiragolumab (anti-TIGIT therapy) in combination with atezolizumab (Tecentriq) was approved by the FDA for metastatic NSCLC with high PD-L1 based on promising clinical evidence [101]. *TSC22D3* expression induction could lead to the modulation of T cell activation and apoptosis [102]. *TSC22D3* was overexpressed in NSCLC tumor B cells and PBL T cells (Figure 2). TSC22D3 was associated with sensitivity to erlotinib and gefitinib (Table 1 and Table 2). *TSC22D3* was associated with a good prognosis in NSCLC patients [103]. *TSC22D3* was frequently present in the T cell state transitions from intermediate to the pre-dysfunction and dysfunction states [103]. Overall, this study conducted a comprehensive analysis of public single-cell RNA sequencing data and identified DE genes in NSCLC PBL and tumor T cells with prognostic and therapeutic importance.

Targeted therapies and repositioning drugs were identified from the CMap database using our novel AI pipeline [43,44] integrating Boolean implication modeling of tumor-specific B cell co-expression networks, DE genes in NSCLC PBLs and tumor T cells, bulk tumor transcriptome and proteome, and in vitro CRISPR-Cas9/RNAi and drug screening in human NSCLC epithelial cell lines. The CMap input genes were differentially expressed in the single-cell RNA sequencing of NSCLC tumor B cells, PBLs, and tumor T cells with prognostic and predictive implications in a comprehensive evaluation of public patient data. Their functional involvement in NSCLC cell proliferation was substantiated with public genome-scale CRISPR-Cas9/RNAi screening data. Rigorous filtering criteria were applied in defining the CMap gene expression signature. Three pro-survival genes in the nine-gene prognostic marker panel (*ANAPC5*, *EWSR1*, and *EXOC4*) were removed from the input list due to their proliferative roles in NSCLC indicated by the CRISPR-Cas9/RNAi screening results. Similarly, genes showing any conflicting results of drug sensitivity or resistance to the 10 studied therapeutic regimens were removed from the CMap list. As a result, the identified targeted therapies and repositioning drugs are highly relevant to upregulating epithelial and drug-sensitive genes and downregulating mesenchymal, drug-resistant, proliferative, and survival hazard genes in NSCLC patient samples. The selected small molecules were further examined based on their public drug response measurements in 135 human CCLE NSCLC epithelial cell lines. Lestaurtinib, TW-37, and danusertib had small average IC_50_ and EC_50_ values without any outliers (IC_50_ or EC_50_ higher than 10 μM) in 135 cell lines, indicating their efficacy in inhibiting NSCLC cell growth with a potentially safe dose. Danusertib showed an inhibitory association with the gene expression of *CD27*, an emerging ICI for solid tumors [75]. TG-101348 had an inhibitory association with *PDL1* gene expression. Out of the four selected compounds, danusertib, TG-101348, and TW-37 have been investigated in pre-clinical and clinical studies for improving NSCLC treatment. Lestaurtinib, a tyrosine kinase inhibitor of *FLT3* for treating AML [80,81], could potentially be repositioned for treating NSCLC based on its ability to potently kill and inhibit NSCLC cells and its high relevance in reversing EMT, enhancing drug response, inhibiting proliferation in NSCLC cells, and downregulating survival hazard genes in NSCLC patients. This AI pipeline can effectively determine the disease relevance of targeted therapies before clinical trials, thereby expediting drug repositioning R&D for pharmaceutical companies. It also provides an efficient tool for oncologists to choose a targeted therapy for refractory NSCLC patients after the failure of initial treatments.

The current single-cell RNA sequencing techniques have not become routine assays for cancer research or clinical testing. Similarly, although CRISPR-Cas9 has been explored in editing immune cells [104,105,106,107], genome-scale CRISPR-Cas9/RNAi screening data are lacking for broad research applications. This study leveraged public data consortia including TCGA and CCLE. The public single-B-cell sequencing data [45] do not have matched patient clinical follow-up information. This dataset [45] was used to identify the single B-cell gene co-expression work (Figure 1) that was missing in tumor B cells. This network consisted of proliferation genes identified from CRISPR-Cas9/RNAi genome-scale screening data of human epithelial NSCLC cell lines. The nine-gene signature was derived from this network, showing prognostic capacity in RNA sequencing profiles of NSCLC bulk tumors. An ideal study design would be using the same patient cohort for single-cell sequencing, CRISPR-Cas9/RNAi screening, and prognostication. Nevertheless, it is not technically feasible for our research group to conduct such studies currently. The presented conceptual framework could be applied in future research when it is feasible to implement this study design with technology advancement.

There was a remarkable difference in the proportion of low-risk patients in the training and validation sets, i.e., 19% vs. 68%. The training set (GSE81089) [52,53] utilized snap-frozen tumor samples in RNA sequencing, whereas the validation set (TCGA) [58] contained mostly formalin-fixed paraffin-embedded (FFPE) samples. The difference in the sample preservation and RNA degradation since fixing is a possible factor contributing to the different RNA expression quantification scales in both datasets. In RNA sequencing data analysis, housekeeping genes are not generally used for normalization as in qRT-PCR. Therefore, the variation in gene expression measurements due to different sample preparation techniques is not accounted for in the RNA expression analysis. The nine-gene prognostic model was unchanged when applied in the training and validation sets. Therefore, the resulting patient stratification in the validation set containing FFPE samples could be skewed because of the variation in the gene expression measurements caused by the different sample preparation technique. This study showed the feasibility of using this nine-gene signature for patient stratification using RNA sequencing of bulk tumors. In future clinical applications, the training model based on this nine-gene panel will need to be calibrated according to a specific manufacturing platform to ensure optimal prognostication.

## 5. Conclusions

NSCLC remains the leading cause of cancer-related mortality, despite the promising results from immunotherapy. There are currently no biomarkers to identify early-stage NSCLC patients of all histology who are at risk for tumor recurrence/metastasis and benefit from adjuvant therapies. There is a pressing demand for predictive biomarkers of immunotherapy in NSCLC. The mechanisms underlying B cell dysfunction and their prognostic significance in NSCLC are not well understood. To meet these critical needs, this study identified a tumor-specific B cell proliferation and prognostic gene co-expression network in NSCLC using Boolean implication modeling of single-cell RNA sequencing data. A nine-gene marker panel within this network provided accurate prognostic stratification for early-stage NSCLC patients using RNA sequencing and proteomic profiles. DE genes in NSCLC tumor B cells, PBLs, and tumor T cells with prognostic implications were garnered. Numerous selected genes had a significant association with radiotherapy response and drug sensitivity/resistance to 10 commonly used NSCLC therapeutic regimens. Based on these results, functional pathways, targeted therapies, and repositioning drugs were discovered using a novel AI pipeline to prolong NSCLC patient survival, improve treatment response, inhibit proliferation, and reverse EMT. These rigorous analyses of extensive public data generated solid results and hypotheses for future clinical investigations and will aid the development of novel therapies to improve NSCLC patient outcomes.

## 6. Patents

This work is included in a US provisional patent application with serial number 63/355353.

## Figures and Tables

**Table 1:** Genes with significant differential expression (*p* < 0.05; two-sample *t*-tests) in mRNA in sensitive vs. resistant NSCLC cell lines (*n* = 135) to the selected regiments. The genes in blue font are drug-sensitive genes which expressed higher in sensitive cell lines; the genes in red font are drug-resistant genes that expressed higher in resistant cell lines.

	GDSC1	GDSC2	PRISM
Carboplatin			***DUSP2***, ***HLA-DRB1***, ***TIGIT***, ***TNIP3***
Cisplatin		***DUSP2***	***ANAPC5***, ***OAS1***, ***VCP***, ***IL2RA***
Docetaxel	***LMNA***, ***PPP2R1A***, ***TNIP3***	***LMNA***	***IL2RA***, ***CCND2***, ***PPP2R1A***
Erlotinib	***DUSP2***, ***TSC22D3***	***HRH1***, ***LMNA***	***HRH1***, ***LMNA***
Etoposide	***CCT3***, ***EWSR1***, ***TUBA1B***		***CCT3***, ***VCP***, ***HSD17B13***
Gefitinib	***VCP***, ***HSD17B13***	***LMNA***, ***MAP4***, ***TUBA1B***	***CCND2***, ***CCT3***, ***HRH1***, ***HSD17B13***
Gemcitabine		***CCT3***	***CD74***, ***DUSP2***, ***HLA-DRA***, ***TIGIT***
Paclitaxel		***DUSP2***, ***VCP***	***OAS1***, ***PPP2R1A***, ***TNIP3***, ***HSD17B13***
Pemetrexed	***CCT3***, ***DUSP2***		
Vinorelbine	***EXOC4***	***CCT3***, ***DUSP2***, ***LMNA***, ***VCP***	***CCND2***, ***DUSP2***, ***HRH1***, ***LMNA***

**Table 2:** Genes with significant differential expression (*p* < 0.05; two-sample *t*-tests) in protein in sensitive vs. resistant NSCLC cell lines (*n* = 63) to the selected regiments. The genes in blue font are drug-sensitive genes that expressed higher in sensitive cell lines; the genes in red font are drug-resistant genes that expressed higher in resistant cell lines.

	GDSC1	GDSC2	PRISM
Carboplatin			
Cisplatin	**LMNA**		**CCT3**, **OAS1**, **VCP**, **PPP2R1A**
Docetaxel	**PPP2R1A**, **TUBA1B**		**GBP4**
Erlotinib		**CCT3**	**VCP**
Etoposide			**LMNA**
Gefitinib	**CCT3**, **HLA-DRA**, **TSC22D3**	**CCT3**	**CCT3**, **OAS1**
Gemcitabine		**EXOC4**	**CD74**, **LMNA**, **VCP**
Paclitaxel			**CD74**, **HLA-DRA**, **TUBA1B**
Pemetrexed	**ANAPC5**, **HRH1**	**ANAPC5**, **HRH1**	
Vinorelbine			

**Table 3:** The significant (*p* < 0.05, connectivity score > 0.9) consensus signatures from overexpression and shRNA knockdown targeted the same genes in NSCLC cell lines in CMap. OE: overexpression assay. KD: knockdown. SH: shRNA assay.

Src_Set_Id	Cell_Name	Pert_Type	Genes
OE_CELL_CYCLE_INHIBITION	A549	TRT_OE	*CDKN1A*, *CDKN1B*, *CDKN2C*
BIOCARTA_AHSP_PATHWAY	A549	TRT_SH.CGS	*AHSP*, *ALAD*, *ALAS1*, *ALAS2*, *CPOX*, *FECH*, *GATA1*, *HBA1*, *HBA2*, *HBB*, *HMBS*, *UROD*, *UROS*
KD_AHSP_PATHWAY	A549	TRT_SH.CGS	*ALAD*, *FECH*, *GATA1*, *UROD*
KD_RIBOSOMAL_40S_SUBUNIT	A549	TRT_SH.CGS	*FAU*, *RPS3*, *RPS3A*, *RPS5*, *RPS6*, *RPS7*, *RPS9*, *RPS10*, *RPS13*, *RPS14*, *RPS15A*, *RPS16*, *RPS19*, *RPS27A*
KEGG_TAURINE_AND_ HYPOTAURINE_METABOLISM	A549	TRT_SH.CGS	*ADO*, *BAAT*, *CDO1*, *CSAD*, *GAD1*, *GAD2*, *GGT1*, *GGT5*, *GGT6*, *GGT7*
KEGG_TERPENOID_ BACKBONE_BIOSYNTHESIS	A549	TRT_SH.CGS	*ACAT1*, *ACAT2*, *DHDDS*, *FDPS*, *GGPS1*, *HMGCR*, *HMGCS1*, *HMGCS2*, *IDI1*, *IDI2*, *MVD*, *MVK*, *PDSS1*, *PDSS2*, *PMVK*
OE_PHOSPHOLIPASES	A549	TRT_SH.CGS	*PLCG2*, *PLA2G12B*, *PLCB1*, *PLD1*
PID_VEGF_VEGFR_PATHWAY	HCC515	TRT_SH.CGS	*FLT1*, *FLT4*, *KDR*, *NRP1*, *NRP2*, *PGF*, *VEGFA*, *VEGFB*, *VEGFC*, *VEGFD*
REACTOME_HOMOLOGOUS_ RECOMBINATION_ REPAIR_OF_REPLICATION_ INDEPENDENT_ DOUBLE_STRAND_BREAKS	A549	TRT_SH.CGS	*ATM*, *BRCA1*, *BRCA2*, *BRIP1*, *H2AX*, *LIG1*, *MDC1*, *MRE11*, *NBN*, *RAD50*, *RAD51*, *RAD52*, *RPA1*, *RPA2*, *RPA3*, *TP53BP1*
REACTOME_HYALURONAN_ METABOLISM	A549	TRT_SH.CGS	*ABCC5*, *CD44*, *CEMIP*, *CHP1*, *GUSB*, *HAS1*, *HAS2*, *HAS3*, *HEXA*, *HEXB*, *HMMR*, *HYAL1*, *HYAL2*, *HYAL3*, *LYVE1*, *SLC9A1*, *STAB2*
REACTOME_HYALURONAN_ UPTAKE_AND_DEGRADATION	A549	TRT_SH.CGS	*CD44*, *CHP1*, *GUSB*, *HEXA*, *HEXB*, *HMMR*, *HYAL1*, *HYAL2*, *HYAL3*, *LYVE1*, *SLC9A1*, *STAB2*
REACTOME_PROCESSIVE_ SYNTHESIS_ON_THE_ LAGGING_STRAND	A549	TRT_SH.CGS	*DNA2*, *FEN1*, *LIG1*, *PCNA*, *POLA1*, *POLA2*, *POLD1*, *POLD2*, *POLD3*, *POLD4*, *PRIM1*, *PRIM2*, *RPA1*, *RPA2*, *RPA3*
REACTOME_ REMOVAL_OF_THE_FLAP_ INTERMEDIATE_FROM_THE_C_STRAND	A549	TRT_SH.CGS	*DNA2*, *FEN1*, *PCNA*, *POLD1*, *POLD2*, *POLD3*, *POLD4*, *RPA1*, *RPA2*, *RPA3*, *WRN*
REACTOME_REPAIR_ SYNTHESIS_FOR_GAP_ FILLING_BY_DNA_POL_IN_TC_NER	A549	TRT_SH.CGS	*PCNA*, *POLD1*, *POLD2*, *POLD3*, *POLD4*, *POLE*, *POLE2*, *RFC2*, *RFC3*, *RFC4*, *RFC5*, *RPA1*, *RPA2*, *RPA3*
REACTOME_SIGNAL_ REGULATORY_ PROTEIN_SIRP_FAMILY_ INTERACTIONS	A549	TRT_SH.CGS	*CD47*, *FYB*, *GRB2*, *PTK2*, *PTK2B*, *PTPN6*, *SIRPA*, *SIRPB1*, *SIRPG*, *SRC*, *TYROBP*
ST_G_ALPHA_I_PATHWAY	A549	TRT_SH.CGS	*AKT1*, *AKT2*, *AKT3*, *ASAH1*, *BRAF*, *CFB*, *DAG1*, *DRD2*, *EGFR*, *EPHB2*, *GRB2*, *ITPKA*, *ITPKB*, *ITPR1*, *ITPR2*, *ITPR3*, *KCNJ3*, *KCNJ5*, *KCNJ9*, *MAPK1*, *PI3*, *PIK3CB*, *PITX2*, *PLCB1*, *PLCB2*, *PLCB3*, *PLCB4*, *RAF1*, *RAP1GAP*, *RGS20*, *SHC1*, *SOS1*, *SOS2*, *SRC*, *STAT3*, *TERF2IP*

## Data Availability

All data included in this analysis are publicly available with data access provided in the manuscript.

## Supplementary Materials

The following supporting information included in Supplementary Files can be downloaded at: www.mdpi.com/article/10.3390/cancers14133123/s1. Supplementary File S1 includes Tables S1–S8 and Figure S1. Table S1: Detailed information on the selected candidate prognostic genes, Table S2: Biological context of network edges in Figure 1A, Table S3: Significantly enriched pathways in the ToppGene enrichment analysis of the B-cell network in Figure 1A, Table S4: The cutoff values to stratify high expression and low expression of genes in Figure 3B,C, Table S5: Detailed information of CMap input gene lists (Figure 5A), Table S6: The significant (*p* < 0.05, connectivity score > 0.9) compounds from CMap, Table S7: Biological context of edges in Supplementary Figure S1A network, Table S8: Biological context of edges in Supplementary Figure S1B network, Figure S1: Tumor-specific B-cell proliferation networks involving PD1 (PDCD1), PDL1 (CD274), and CTLA4 in NSCLC, File S2: The normalized dependency scores of proliferation genes (Figure 1A) in RNAi screening and in CRISPR-Cas9 screening, File S3: Details of TCGA validation of the 9-gene predictive signature.
