# Supplementary material for: Single B Cell Gene Co-Expression Networks Implicated in Prognosis, Proliferation, and Therapeutic Responses in Non-Small Cell Lung Cancer Bulk Tumors
Source: Cancers (Basel). 2022 Jun 25;14(13):3123. doi: 10.3390/cancers14133123 (PMC9265014; doi:10.3390/cancers14133123)
Supplement: Supplementary file 1 [file cancers-14-03123-s001.zip › Supplementary file S1.pdf]

**Supplementary Table S1.** Detailed information on the selected candidate prognostic genes.

| Gene | Cox model in NSCLC tumor RNA-seq data (GSE81089) | | | | Results of *t*-tests in NSCLC tumor microarray data (GSE28582) | |
|---|---|---|---|---|---|---|
| | **Cutoff Point** | ***p*-value** | **HR** | **95%CI** | **Fold Change (short-survival/long-survival)** | ***p*-value** |
| ADCY2 | 0.421 | 0.016 | 0.598 | [0.3928, 0.9092] | 0.929 | 0.013 |
| ARGLU1 | 66.804 | 0.00085 | 0.491 | [0.3238, 0.7459] | 0.977 | 0.044 |
| ARNTL2 | 11.982 | 0.0045 | 1.852 | [1.2105, 2.8333] | 1.084 | 0.0099 |
| EXOC4 | 27.970 | 0.025 | 1.731 | [1.0724, 2.7928] | 1.018 | 0.028 |
| FAM118A | 32.030 | 0.026 | 0.621 | [0.409, 0.944] | 0.965 | 0.047 |
| FGF5 | 0.179 | 0.015 | 1.762 | [1.118, 2.779] | 1.024 | 0.012 |
| HRH1 | 5.977 | 9.02E-05 | 2.262 | [1.503, 3.403] | 1.051 | 0.0097 |
| KRR1 | 76.966 | 0.021 | 1.917 | [1.102, 3.336] | 1.026 | 0.034 |
| LONRF2 | 0.696 | 0.011 | 0.588 | [0.390, 0.886] | 0.918 | 0.048 |
| MAP4 | 43.202 | 0.0043 | 2.731 | [1.371, 5.440] | 1.019 | 0.017 |
| MAP4K4 | 52.814 | 0.0013 | 1.977 | [1.305, 2.996] | 1.025 | 0.039 |
| PTBP3 | 41.805 | 0.012 | 1.696 | [1.121, 2.565] | 1.015 | 0.035 |
| RSL24D1 | 53.082 | 0.027 | 1.622 | [1.057, 2.490] | 1.019 | 0.050 |
| TP53INP1 | 20.588 | 0.0049 | 0.521 | [0.331, 0.820] | 0.966 | 0.022 |
| TRIM38 | 11.492 | 0.026 | 0.533 | [0.307, 0.926] | 0.975 | 0.024 |
| TSPYL2 | 18.323 | 0.0016 | 0.501 | [0.326, 0.770] | 0.979 | 0.012 |
| UBL5 | 205.301 | 0.0061 | 0.887 | [0.482, 1.631] | 0.986 | 0.025 |
| WDR5B | 3.354 | 0.0028 | 0.466 | [0.283, 0.769] | 0.970 | 0.019 |

**Supplementary Table S2.** Biological context of network edges in Figure 1A (Precision Threshold: 0.7704, Scope Threshold: 0.1462; *p*-value < 0.00005; one-tailed *z*-tests).

| Biological context | Precision | Scope |
|---|---|---|
| The up-regulation of ACTR3 implies the up-regulation of RPL37. | 0.869388 | 0.159505 |
| The up-regulation of ANAPC5 implies the up-regulation of ITPRIPL2. | 0.794872 | 0.152344 |
| The up-regulation of APOC1P1 implies the up-regulation of PNPT1. | 0.847619 | 0.205078 |
| The up-regulation of APOC1P1 implies the up-regulation of RPL37. | 0.934694 | 0.159505 |
| The up-regulation of ARCN1 implies the up-regulation of LOC100505876. | 0.788235 | 0.147569 |
| The up-regulation of ARCN1 implies the up-regulation of ORC6. | 0.782805 | 0.19184 |
| The up-regulation of ARCN1 implies the up-regulation of PNPT1. | 0.87451 | 0.166016 |
| The up-regulation of ARCN1 implies the up-regulation of RPP30. | 0.823529 | 0.177083 |
| The up-regulation of ARGLU1 implies the up-regulation of EEF2. | 0.896104 | 0.200521 |
| The up-regulation of ARGLU1 implies the up-regulation of KMT2E. | 0.857708 | 0.219618 |
| The up-regulation of ARGLU1 implies the up-regulation of RPL27. | 0.875325 | 0.167101 |
| The up-regulation of ARGLU1 implies the up-regulation of RPL37. | 0.937662 | 0.167101 |
| The up-regulation of ARGLU1 implies the up-regulation of RPL37A. | 1 | 0.162326 |
| The up-regulation of ARGLU1 implies the up-regulation of RPS21. | 0.945455 | 0.190972 |
| The up-regulation of ARGLU1 implies the up-regulation of RPS29. | 0.937662 | 0.167101 |
| The up-regulation of ARNTL2 implies the up-regulation of ITPRIPL2. | 0.776224 | 0.186198 |
| The up-regulation of ARNTL2 implies the up-regulation of PNPT1. | 0.870707 | 0.161133 |
| The up-regulation of BLK implies the up-regulation of EEF2. | 0.832753 | 0.186849 |

| | | |
|---|---|---|
| The up-regulation of BLK implies the up-regulation of RPL27. | 0.933101 | 0.155707 |
| The up-regulation of BLK implies the up-regulation of RPL37. | 1 | 0.155707 |
| The up-regulation of BLK implies the up-regulation of RPL37A. | 0.931133 | 0.151259 |
| The up-regulation of BLK implies the up-regulation of RPS21. | 0.941463 | 0.177951 |
| The up-regulation of BLK implies the up-regulation of RPS29. | 0.933101 | 0.155707 |
| The up-regulation of BLK implies the up-regulation of SON. | 0.804878 | 0.213542 |
| The up-regulation of CCT3 implies the up-regulation of PNPT1. | 0.793548 | 0.151367 |
| The up-regulation of CES2 implies the up-regulation of PNPT1. | 0.80155 | 0.209961 |
| The up-regulation of CES2 implies the up-regulation of RPL37. | 0.808638 | 0.163303 |
| The up-regulation of CNOT1 implies the up-regulation of EEF2. | 0.850932 | 0.209635 |
| The up-regulation of CNOT1 implies the up-regulation of RPL27. | 0.940373 | 0.174696 |
| The up-regulation of CNOT1 implies the up-regulation of RPL37. | 0.880745 | 0.174696 |
| The up-regulation of CNOT1 implies the up-regulation of RPL37A. | 0.877238 | 0.169705 |
| The up-regulation of CNOT1 implies the up-regulation of RPS21. | 0.843478 | 0.199653 |
| The up-regulation of CNOT1 implies the up-regulation of RPS29. | 0.821118 | 0.174696 |
| The up-regulation of DDX3X implies the up-regulation of EEF2. | 0.805471 | 0.214193 |
| The up-regulation of DDX3X implies the up-regulation of RPL37. | 0.883283 | 0.178494 |
| The up-regulation of DDX3X implies the up-regulation of RPL37A. | 0.87985 | 0.173394 |
| The up-regulation of DDX3X implies the up-regulation of RPS21. | 0.846809 | 0.203993 |
| The up-regulation of DDX3X implies the up-regulation of RPS29. | 0.883283 | 0.178494 |
| The up-regulation of DDX46 implies the up-regulation of RPL37. | 0.859341 | 0.148112 |
| The up-regulation of DDX46 implies the up-regulation of RPS29. | 0.789011 | 0.148112 |
| The up-regulation of DDX6 implies the up-regulation of EEF2. | 0.900621 | 0.209635 |
| The up-regulation of DDX6 implies the up-regulation of RPL27. | 1 | 0.174696 |
| The up-regulation of DDX6 implies the up-regulation of RPL37. | 1 | 0.174696 |
| The up-regulation of DDX6 implies the up-regulation of RPL37A. | 1 | 0.169705 |
| The up-regulation of DDX6 implies the up-regulation of RPS21. | 0.947826 | 0.199653 |
| The up-regulation of DDX6 implies the up-regulation of RPS29. | 1 | 0.174696 |
| The up-regulation of EEF2 implies the up-regulation of RPL27. | 0.847619 | 0.205078 |
| The up-regulation of EEF2 implies the up-regulation of RPL37. | 0.949206 | 0.205078 |
| The up-regulation of EEF2 implies the up-regulation of RPL37A. | 0.895425 | 0.199219 |
| The up-regulation of EEF2 implies the up-regulation of RPS21. | 0.822222 | 0.234375 |
| The up-regulation of EEF2 implies the up-regulation of RPS29. | 0.898413 | 0.205078 |
| The up-regulation of EIF2S3 implies the up-regulation of RPL27. | 0.776093 | 0.186089 |
| The up-regulation of EIF2S3 implies the up-regulation of RPL37. | 0.776093 | 0.186089 |
| The up-regulation of EIF3E implies the up-regulation of RPL27. | 0.866202 | 0.155707 |
| The up-regulation of EIF3E implies the up-regulation of RPL37. | 0.933101 | 0.155707 |
| The up-regulation of EIF3E implies the up-regulation of RPL37A. | 0.7934 | 0.151259 |
| The up-regulation of EIF3E implies the up-regulation of RPS29. | 0.799303 | 0.155707 |
| The up-regulation of EIF3H implies the up-regulation of RPL27. | 0.808638 | 0.163303 |
| The up-regulation of EIF3H implies the up-regulation of RPL37. | 0.808638 | 0.163303 |
| The up-regulation of EIF3H implies the up-regulation of RPL37A. | 0.80301 | 0.158637 |
| The up-regulation of EIF3H implies the up-regulation of RPS21. | 0.82558 | 0.186632 |
| The up-regulation of EIF3H implies the up-regulation of RPS29. | 0.808638 | 0.163303 |
| The up-regulation of EIF4E implies the up-regulation of CES2. | 0.792992 | 0.20128 |

| | | |
|---|---|---|
| The up-regulation of EIF4E implies the up-regulation of LOC100505876. | 0.794286 | 0.15191 |
| The up-regulation of EWSR1 implies the up-regulation of EEF2. | 0.832753 | 0.186849 |
| The up-regulation of EWSR1 implies the up-regulation of PNPT1. | 0.843902 | 0.200195 |
| The up-regulation of EWSR1 implies the up-regulation of RPL27. | 0.866202 | 0.155707 |
| The up-regulation of EWSR1 implies the up-regulation of RPL37. | 1 | 0.155707 |
| The up-regulation of EWSR1 implies the up-regulation of RPL37A. | 0.931133 | 0.151259 |
| The up-regulation of EWSR1 implies the up-regulation of RPS29. | 0.933101 | 0.155707 |
| The up-regulation of EXOC4 implies the up-regulation of ARGLU1. | 0.872679 | 0.163628 |
| The up-regulation of EXOC4 implies the up-regulation of WDR5B. | 0.805274 | 0.160482 |
| The up-regulation of FAM118A implies the up-regulation of CES2. | 0.791001 | 0.149523 |
| The up-regulation of HNRNPU implies the up-regulation of EEF2. | 0.819549 | 0.173177 |
| The up-regulation of HRH1 implies the up-regulation of LINC00907. | 0.868132 | 0.157986 |
| The up-regulation of HUWE1 implies the up-regulation of RPS21. | 0.810526 | 0.164931 |
| The up-regulation of ITPRIPL2 implies the up-regulation of PNPT1. | 0.90303 | 0.214844 |
| The up-regulation of ITPRIPL2 implies the up-regulation of RPL37. | 0.812987 | 0.167101 |
| The up-regulation of KMT2E implies the up-regulation of RPL27. | 0.945143 | 0.189887 |
| The up-regulation of KMT2E implies the up-regulation of RPL37. | 0.890286 | 0.189887 |
| The up-regulation of KMT2E implies the up-regulation of RPL37A. | 0.943529 | 0.184462 |
| The up-regulation of KMT2E implies the up-regulation of RPS21. | 0.904 | 0.217014 |
| The up-regulation of KMT2E implies the up-regulation of RPS29. | 0.945143 | 0.189887 |
| The up-regulation of KRR1 implies the up-regulation of NCL. | 0.928994 | 0.146701 |
| The up-regulation of LINC00907 implies the up-regulation of RPL37. | 0.812987 | 0.167101 |
| The up-regulation of LONRF2 implies the up-regulation of LOC100505876. | 0.776744 | 0.186632 |
| The up-regulation of MAP4 implies the up-regulation of LOC100505876. | 0.87027 | 0.16059 |
| The up-regulation of MAP4 implies the up-regulation of RPP30. | 0.783784 | 0.192708 |
| The up-regulation of MAP4K4 implies the up-regulation of TRIM22. | 0.786667 | 0.146484 |
| The up-regulation of MDN1 implies the up-regulation of EIF3E. | 0.831085 | 0.185004 |
| The up-regulation of NAA50 implies the up-regulation of PNPT1. | 0.796825 | 0.205078 |
| The up-regulation of NAA50 implies the up-regulation of RPL37. | 0.804082 | 0.159505 |
| The up-regulation of NCL implies the up-regulation of RPL27. | 0.812987 | 0.167101 |
| The up-regulation of NCL implies the up-regulation of RPL37. | 0.937662 | 0.167101 |
| The up-regulation of NCL implies the up-regulation of RPL37A. | 0.871658 | 0.162326 |
| The up-regulation of NCL implies the up-regulation of RPS21. | 0.836364 | 0.190972 |
| The up-regulation of NCL implies the up-regulation of RPS29. | 0.875325 | 0.167101 |
| The up-regulation of NCL implies the up-regulation of SON. | 0.772727 | 0.229167 |
| The up-regulation of NUP98 implies the up-regulation of EEF2. | 0.819549 | 0.173177 |
| The up-regulation of PNPT1 implies the up-regulation of RPL37. | 0.892437 | 0.193685 |
| The up-regulation of PPP2R1A implies the up-regulation of ACTR3. | 0.857778 | 0.146484 |
| The up-regulation of PSMA3 implies the up-regulation of EEF2. | 0.792208 | 0.150391 |
| The up-regulation of PTBP3 implies the up-regulation of ORC6. | 0.794872 | 0.152344 |
| The up-regulation of RPL37 implies the up-regulation of RPS29, and the nonup-regulation of RPL37 implies the nonup-regulation of RPS29. | 0.775176 | 0.185124 |
| The up-regulation of RPL37A implies the up-regulation of RPL27, and the nonup-regulation of RPL37A implies the nonup-regulation of RPL27. | 0.796418 | 0.1535 |
| The up-regulation of RPL37A implies the up-regulation of RPS29, and the nonup-regulation of RPL37A implies the nonup-regulation of RPS29. | 0.796418 | 0.1535 |

| | | |
|---|---|---|
| The up-regulation of RPP30 implies the up-regulation of LOC100505876. | 0.8 | 0.208333 |
| The up-regulation of RPS21 implies the up-regulation of RPL27. | 0.902041 | 0.212674 |
| The up-regulation of RPS21 implies the up-regulation of RPL37. | 0.804082 | 0.212674 |
| The up-regulation of RPS21 implies the up-regulation of RPL37A. | 0.94958 | 0.206597 |
| The up-regulation of RPS21 implies the up-regulation of RPS29. | 0.853061 | 0.212674 |
| The up-regulation of RSL24D1 implies the up-regulation of DDX3X. | 0.797326 | 0.154188 |
| The up-regulation of SF3A3 implies the up-regulation of APOC1P1. | 0.816092 | 0.169922 |
| The up-regulation of SF3B1 implies the up-regulation of RPS29. | 0.828571 | 0.182292 |
| The up-regulation of SON implies the up-regulation of EEF2. | 0.809524 | 0.21875 |
| The up-regulation of SON implies the up-regulation of RPL27. | 0.828571 | 0.182292 |
| The up-regulation of SON implies the up-regulation of RPL37. | 1 | 0.182292 |
| The up-regulation of SON implies the up-regulation of RPL37A. | 0.882353 | 0.177083 |
| The up-regulation of SON implies the up-regulation of RPS21. | 0.85 | 0.208333 |
| The up-regulation of SON implies the up-regulation of RPS29. | 0.942857 | 0.182292 |
| The up-regulation of SRSF1 implies the up-regulation of ARGLU1. | 0.794872 | 0.152344 |
| The up-regulation of SUPT5H implies the up-regulation of ARGLU1. | 0.857988 | 0.146701 |
| The up-regulation of TP53INP1 implies the up-regulation of KMT2E. | 0.804348 | 0.159722 |
| The up-regulation of TRIM22 implies the up-regulation of EEF2. | 0.782313 | 0.191406 |
| The up-regulation of TRIM22 implies the up-regulation of RPL27. | 0.804082 | 0.159505 |
| The up-regulation of TRIM22 implies the up-regulation of RPL37. | 0.869388 | 0.159505 |
| The up-regulation of TRIM22 implies the up-regulation of RPL37A. | 0.865546 | 0.154948 |
| The up-regulation of TRIM22 implies the up-regulation of RPS21. | 0.885714 | 0.182292 |
| The up-regulation of TRIM22 implies the up-regulation of RPS29. | 0.869388 | 0.159505 |
| The up-regulation of TRIM38 implies the up-regulation of RPL37. | 0.872425 | 0.163303 |
| The up-regulation of TSPYL2 implies the up-regulation of BLK. | 0.790545 | 0.149197 |
| The up-regulation of TUBA1B implies the up-regulation of DDX6. | 0.82 | 0.173611 |
| The up-regulation of UBA1 implies the up-regulation of NCL. | 0.815385 | 0.169271 |
| The up-regulation of UBA1 implies the up-regulation of PNPT1. | 0.786667 | 0.146484 |
| The up-regulation of UBA1 implies the up-regulation of SON. | 0.933333 | 0.15625 |
| The up-regulation of UBE2I implies the up-regulation of LOC100505876. | 0.805405 | 0.16059 |
| The up-regulation of UBL5 implies the up-regulation of EEF2. | 0.930736 | 0.150391 |
| The up-regulation of URB1 implies the up-regulation of CES2. | 0.798742 | 0.155273 |
| The up-regulation of VCP implies the up-regulation of NCL. | 0.794872 | 0.152344 |
| The up-regulation of WBP11 implies the up-regulation of DDX6. | 0.786667 | 0.146484 |
| The up-regulation of WDR5B implies the up-regulation of LOC100505876. | 0.84 | 0.195312 |
| The up-regulation of WDR5B implies the up-regulation of RPL37. | 0.817143 | 0.170898 |
| The up-regulation of ZNF207 implies the up-regulation of KMT2E. | 0.804348 | 0.159722 |
| The up-regulation of ZNF207 implies the up-regulation of PNPT1. | 0.8 | 0.15625 |

**Supplementary Table S3.** Significantly enriched pathways in the ToppGene enrichment analysis of the B-cell network in Figure 1A.

| ID | Name | Source | *p*-value | *q*-value Bonferroni | *q*-value FDR B&H | *q*-value FDR B&Y | Hit Count in Query List | Hit Count in Genome | Hit in Query List |
|---|---|---|---|---|---|---|---|---|---|
| 1269649 | Gene Expression | BioSystems: REACTOME | 2.20E-10 | 1.80E-07 | 5.62E-08 | 4.09E-07 | 25 | 1844 | RPL27,PPP2R1A,WBP11,EEF2,DDX46,RPL37,RPL37A,SRSF1,SF3B1,TP53INP1,UBE2I,SUPT5H,EIF2S3,PSMA3,EIF4E,RPP30,EIF3E,NUP98,SF3A3,RPS21,EIF3H,RPS29,KRR1,CNOT1,HNRNPU |
| 1268678 | Translation | BioSystems: REACTOME | 2.27E-10 | 1.86E-07 | 5.62E-08 | 4.09E-07 | 10 | 165 | RPL27,EEF2,RPL37,RPL37A,EIF2S3,EIF4E,EIF3E,RPS21,EIF3H,RPS29 |
| 1268686 | GTP hydrolysis and joining of the 60S ribosomal subunit | BioSystems: REACTOME | 2.74E-10 | 2.25E-07 | 5.62E-08 | 4.09E-07 | 9 | 119 | RPL27,RPL37,RPL37A,EIF2S3,EIF4E,EIF3E,RPS21,EIF3H,RPS29 |
| 1268688 | L13a-mediated translational silencing of Ceruloplasmin expression | BioSystems: REACTOME | 2.74E-10 | 2.25E-07 | 5.62E-08 | 4.09E-07 | 9 | 119 | RPL27,RPL37,RPL37A,EIF2S3,EIF4E,EIF3E,RPS21,EIF3H,RPS29 |
| 1268680 | Cap-dependent Translation Initiation | BioSystems: REACTOME | 4.92E-10 | 4.03E-07 | 6.72E-08 | 4.90E-07 | 9 | 127 | RPL27,RPL37,RPL37A,EIF2S3,EIF4E,EIF3E,RPS21,EIF3H,RPS29 |
| 1268679 | Eukaryotic Translation Initiation | BioSystems: REACTOME | 4.92E-10 | 4.03E-07 | 6.72E-08 | 4.90E-07 | 9 | 127 | RPL27,RPL37,RPL37A,EIF2S3,EIF4E,EIF3E,RPS21,EIF3H,RPS29 |
| 1268685 | Ribosomal scanning and start codon recognition | BioSystems: REACTOME | 8.08E-08 | 6.61E-05 | 8.00E-06 | 5.83E-05 | 6 | 63 | EIF2S3,EIF4E,EIF3E,RPS21,EIF3H,RPS29 |
| 1268681 | Formation of a pool of free 40S subunits | BioSystems: REACTOME | 8.53E-08 | 6.99E-05 | 8.00E-06 | 5.83E-05 | 7 | 107 | RPL27,RPL37,RPL37A,EIF3E,RPS21,EIF3H,RPS29 |
| 1268684 | Translation initiation complex formation | BioSystems: REACTOME | 8.89E-08 | 7.28E-05 | 8.00E-06 | 5.83E-05 | 6 | 64 | EIF2S3,EIF4E,EIF3E,RPS21,EIF3H,RPS29 |
| 1268683 | Activation of the mRNA upon binding of the cap-binding complex and eIFs, and subsequent binding to 43S | BioSystems: REACTOME | 9.76E-08 | 8.00E-05 | 8.00E-06 | 5.83E-05 | 6 | 65 | EIF2S3,EIF4E,EIF3E,RPS21,EIF3H,RPS29 |
| 1268677 | Metabolism of proteins | BioSystems: REACTOME | 1.95E-07 | 1.60E-04 | 1.46E-05 | 1.06E-04 | 20 | 1631 | TUBA1B,RPL27,EEF2,UBA1,RPL37,RPL37A,UBE2I,CCT3,EIF2S3,PSMA3,EIF4E,TSPYL2,EIF3E,NUP98,RPS21,EIF3H,RPS29,ARCN1,VCP,EXOC4 |
| M189 | Ribosome | MSigDB C2 BIOCARTA (v7.3) | 6.03E-07 | 4.94E-04 | 4.11E-05 | 3.00E-04 | 6 | 88 | RPL27,RPL37,RPL37A,RPS21,RPS29,RSL24D1 |
| 1268691 | Peptide chain elongation | BioSystems: REACTOME | 8.37E-07 | 6.85E-04 | 5.27E-05 | 3.84E-04 | 6 | 93 | RPL27,EEF2,RPL37,RPL37A,RPS21,RPS29 |
| M39423 | Translation Factors | MSigDB C2 BIOCARTA (v7.3) | 1.11E-06 | 9.07E-04 | 6.23E-05 | 4.54E-04 | 5 | 53 | EEF2,EIF2S3,EIF4E,EIF3E,EIF3H |
| 1268690 | Eukaryotic Translation Elongation | BioSystems: REACTOME | 1.14E-06 | 9.34E-04 | 6.23E-05 | 4.54E-04 | 6 | 98 | RPL27,EEF2,RPL37,RPL37A,RPS21,RPS29 |

| | | | | | | | | | |
|---|---|---|---|---|---|---|---|---|---|
| 1268682 | Formation of the ternary complex, and subsequently, the 43S complex | BioSystems: REACTOME | 1.34E-06 | 1.09E-03 | 6.83E-05 | 4.98E-04 | 5 | 55 | EIF2S3,EIF3E,RPS21,EIF3H,RPS29 |
| 177876 | RNA transport | BioSystems: KEGG | 2.07E-06 | 1.70E-03 | 9.85E-05 | 7.17E-04 | 7 | 171 | UBE2I,EIF2S3,EIF4E,RPP30,EIF3E,NUP98,EIF3H |
| 1269688 | Processing of Capped Intron-Containing Pre-mRNA | BioSystems: REACTOME | 2.16E-06 | 1.77E-03 | 9.85E-05 | 7.17E-04 | 8 | 248 | WBP11,DDX46,SRSF1,SF3B1,EIF4E,NUP98,SF3A3,HNRNPU |
| 1383086 | Major pathway of rRNA processing in the nucleolus and cytosol | BioSystems: REACTOME | 3.02E-06 | 2.47E-03 | 1.30E-04 | 9.49E-04 | 7 | 181 | RPL27,RPL37,RPL37A,RPP30,RPS21,RPS29,KRR1 |
| 1269716 | Nonsense-Mediated Decay (NMD) | BioSystems: REACTOME | 3.92E-06 | 3.21E-03 | 1.53E-04 | 1.11E-03 | 6 | 121 | RPL27,PPP2R1A,RPL37,RPL37A,RPS21,RPS29 |
| 1269717 | Nonsense Mediated Decay (NMD) enhanced by the Exon Junction Complex (EJC) | BioSystems: REACTOME | 3.92E-06 | 3.21E-03 | 1.53E-04 | 1.11E-03 | 6 | 121 | RPL27,PPP2R1A,RPL37,RPL37A,RPS21,RPS29 |
| 1427846 | rRNA processing in the nucleus and cytosol | BioSystems: REACTOME | 4.62E-06 | 3.79E-03 | 1.72E-04 | 1.25E-03 | 7 | 193 | RPL27,RPL37,RPL37A,RPP30,RPS21,RPS29,KRR1 |
| M2044 | Spliceosome | MSigDB C2 BIOCARTA (v7.3) | 5.19E-06 | 4.25E-03 | 1.85E-04 | 1.35E-03 | 6 | 127 | WBP11,DDX46,SRSF1,SF3B1,SF3A3,HNRNPU |
| 1383085 | rRNA processing | BioSystems: REACTOME | 6.45E-06 | 5.28E-03 | 2.20E-04 | 1.60E-03 | 7 | 203 | RPL27,RPL37,RPL37A,RPP30,RPS21,RPS29,KRR1 |
| 125136 | Spliceosome | BioSystems: KEGG | 7.07E-06 | 5.79E-03 | 2.32E-04 | 1.69E-03 | 6 | 134 | WBP11,DDX46,SRSF1,SF3B1,SF3A3,HNRNPU |
| 1269056 | Infectious disease | BioSystems: REACTOME | 7.80E-06 | 6.39E-03 | 2.46E-04 | 1.79E-03 | 9 | 393 | RPL27,EEF2,RPL37,RPL37A,SUPT5H,PSMA3,NUP98,RPS21,RPS29 |
| 1269115 | Influenza Viral RNA Transcription and Replication | BioSystems: REACTOME | 8.37E-06 | 6.86E-03 | 2.54E-04 | 1.85E-03 | 6 | 138 | RPL27,RPL37,RPL37A,NUP98,RPS21,RPS29 |
| 1269109 | Influenza Life Cycle | BioSystems: REACTOME | 1.20E-05 | 9.85E-03 | 3.52E-04 | 2.56E-03 | 6 | 147 | RPL27,RPL37,RPL37A,NUP98,RPS21,RPS29 |
| M39495 | Cytoplasmic Ribosomal Proteins | MSigDB C2 BIOCARTA (v7.3) | 1.54E-05 | 1.26E-02 | 4.28E-04 | 3.12E-03 | 5 | 90 | RPL27,RPL37,RPL37A,RPS21,RPS29 |
| 83036 | Ribosome | BioSystems: KEGG | 1.57E-05 | 1.28E-02 | 4.28E-04 | 3.12E-03 | 6 | 154 | RPL27,RPL37,RPL37A,RPS21,RPS29,RSL24D1 |
| 1269120 | Viral mRNA Translation | BioSystems: REACTOME | 1.81E-05 | 1.48E-02 | 4.64E-04 | 3.38E-03 | 5 | 93 | RPL27,RPL37,RPL37A,RPS21,RPS29 |
| 1269108 | Influenza Infection | BioSystems: REACTOME | 1.81E-05 | 1.49E-02 | 4.64E-04 | 3.38E-03 | 6 | 158 | RPL27,RPL37,RPL37A,NUP98,RPS21,RPS29 |
| 1339156 | Selenocysteine synthesis | BioSystems: REACTOME | 2.11E-05 | 1.73E-02 | 5.21E-04 | 3.79E-03 | 5 | 96 | RPL27,RPL37,RPL37A,RPS21,RPS29 |
| 1268692 | Eukaryotic Translation Termination | BioSystems: REACTOME | 2.21E-05 | 1.81E-02 | 5.21E-04 | 3.79E-03 | 5 | 97 | RPL27,RPL37,RPL37A,RPS21,RPS29 |
| M7721 | Eukaryotic protein translation | MSigDB C2 BIOCARTA (v7.3) | 2.23E-05 | 1.82E-02 | 5.21E-04 | 3.79E-03 | 3 | 16 | EEF2,EIF2S3,EIF4E |

| | | | | | | | | | |
|---|---|---|---|---|---|---|---|---|---|
| 1269718 | Nonsense Mediated Decay (NMD) independent of the Exon Junction Complex (EJC) | BioSystems: REACTOME | 2.57E-05 | 2.10E-02 | 5.84E-04 | 4.25E-03 | 5 | 100 | RPL27,RPL37,RPL37A,RPS21,RPS29 |
| M40040 | nsp1 from SARS-CoV-2 inhibits translation initiation in the host cell | MSigDB C2 BIOCARTA (v7.3) | 2.70E-05 | 2.21E-02 | 5.97E-04 | 4.35E-03 | 3 | 17 | EIF2S3,EIF3E,EIF3H |
| 1268854 | Disease | BioSystems: REACTOME | 3.37E-05 | 2.76E-02 | 7.26E-04 | 5.29E-03 | 12 | 867 | RPL27,PPP2R1A,EEF2,RPL37,RPL37A,SUPT5H,PSMA3,NUP98,FGF5,RPS21,RPS29,VCP |
| 1269690 | mRNA Splicing - Major Pathway | BioSystems: REACTOME | 4.83E-05 | 3.95E-02 | 1.01E-03 | 7.39E-03 | 6 | 188 | WBP11,DDX46,SRSF1,SF3B1,SF3A3,HNRNPU |
| 1268689 | SRP-dependent cotranslational protein targeting to membrane | BioSystems: REACTOME | 5.24E-05 | 4.29E-02 | 1.07E-03 | 7.82E-03 | 5 | 116 | RPL27,RPL37,RPL37A,RPS21,RPS29 |
| 1269689 | mRNA Splicing | BioSystems: REACTOME | 6.09E-05 | 4.99E-02 | 1.22E-03 | 8.86E-03 | 6 | 196 | WBP11,DDX46,SRSF1,SF3B1,SF3A3,HNRNPU |
| 1339149 | Selenoamino acid metabolism | BioSystems: REACTOME | 6.93E-05 | 5.68E-02 | 1.35E-03 | 9.85E-03 | 5 | 123 | RPL27,RPL37,RPL37A,RPS21,RPS29 |
| M39406 | mRNA Processing | MSigDB C2 BIOCARTA (v7.3) | 1.01E-04 | 8.23E-02 | 1.91E-03 | 1.39E-02 | 5 | 133 | SRSF1,SF3B1,SUPT5H,SF3A3,HNRNPU |
| M5374 | SARS Coronavirus Protease | MSigDB C2 BIOCARTA (v7.3) | 2.52E-04 | 2.07E-01 | 4.70E-03 | 3.42E-02 | 2 | 7 | EIF4E,NCL |
| M15247 | Ubiquitin mediated proteolysis | MSigDB C2 BIOCARTA (v7.3) | 1.28E-03 | 1.00E+00 | 2.33E-02 | 1.70E-01 | 4 | 135 | UBA1,UBE2I,HUWE1,ANAPC5 |
| 83056 | Ubiquitin mediated proteolysis | BioSystems: KEGG | 1.35E-03 | 1.00E+00 | 2.41E-02 | 1.75E-01 | 4 | 137 | UBA1,UBE2I,HUWE1,ANAPC5 |
| 1270158 | Metabolism of amino acids and derivatives | BioSystems: REACTOME | 1.73E-03 | 1.00E+00 | 3.02E-02 | 2.20E-01 | 6 | 367 | RPL27,RPL37,RPL37A,PSMA3,RPS21,RPS29 |
| 1470929 | Protein methylation | BioSystems: REACTOME | 2.00E-03 | 1.00E+00 | 3.28E-02 | 2.39E-01 | 2 | 19 | EEF2,VCP |
| M194 | Proteasome Complex | MSigDB C2 BIOCARTA (v7.3) | 2.00E-03 | 1.00E+00 | 3.28E-02 | 2.39E-01 | 2 | 19 | UBA1,PSMA3 |
| M16991 | Skeletal muscle hypertrophy is regulated via AKT/mTOR pathway | MSigDB C2 BIOCARTA (v7.3) | 2.00E-03 | 1.00E+00 | 3.28E-02 | 2.39E-01 | 2 | 19 | EIF2S3,EIF4E |

**Supplementary Table S4.** The cutoff values to stratify high expression and low expression of genes in Figure 3B-C.

| gene | TCGA-LUAD & TCGA-LUSC | LUAD Proteomic |
|---|---|---|
| HLA-DRA | 15.76 | 8.87 |
| HLA-DRB1 | 13.36 | 7.76 |
| OAS1 | 11.44 | 6.88 |
| CD74 | 16.55 | 7.95 |

**Supplementary Table S5.** Detailed information of CMap input gene lists (Figure 5A).

| | gene | category |
|---|---|---|
| up gene list | CCND2 | Drug sensitive gene |
| | CD24 | Epithelial gene |
| | CDH1 | Epithelial gene |
| | CLDN7 | Epithelial gene |
| | DDR1 | Epithelial gene |
| | EPCAM | Epithelial gene |
| | ESRP1 | Epithelial gene |
| | ESRP2 | Epithelial gene |
| | IL2RA | Drug sensitive gene |
| | KRT19 | Epithelial gene |
| | RAB25 | Epithelial gene |
| | TIGIT | Drug sensitive gene |
| | TSC22D3 | Drug sensitive gene |
| down gene list | ARCN1 | Proliferation gene |
| | CDK7 | Drug resistant gene |
| | EEF2 | Proliferation gene |
| | FN1 | Mesenchymal gene |
| | HLA-DRB1 | Drug resistant gene |
| | HRH1 | Hazard gene in the 9-gene model |
| | MAP4 | Hazard gene in the 9-gene model / Drug resistant gene |
| | PPP2R1A | Hazard gene in the 9-gene model |
| | PSMA3 | Proliferation gene |
| | SF3B1 | Proliferation gene |
| | SRSF1 | Proliferation gene |
| | TUBA1B | Hazard gene in the 9-gene model |
| | VCP | Hazard gene in the 9-gene model / Proliferation gene |
| | VIM | Mesenchymal gene |
| | ZEB1 | Mesenchymal gene |

**Supplementary Table S6.** The significant ($p < 0.05$, connectivity score > 0.9) compounds from CMap.

| src_set_id | cell_iname | pert_type | compounds |
|---|---|---|---|
| CALCINEURIN_INHIBITOR | A549 | TRT_CP | INCA-6, tacrolimus, cyclosporin-a, tacrolimus |
| CORTICOSTEROID_AGONIST | HCC515 | TRT_CP | dexamethasone, diflorasone, fluocinolone, hydrocortisone, triamcinolone |
| CP_BCL_INHIBITOR | HCC515 | TRT_CP | BH3I-1, BCL2-inhibitor, gossypol, ABT-737, navitoclax, TW-37, obatoclax, HA-14-1 |
| CP_FGFR_INHIBITOR | A549 | TRT_CP | PD-173074, dovitinib, brivanib, orantinib |
| CP_JAK_INHIBITOR | HCC515 | TRT_CP | JAK3-inhibitor-I, JAK3-Inhibitor-II, JAK3-inhibitor-VI, TG-101348, lestaurtinib |
| CP_T_TYPE_CALCIUM_CHANNEL_BLOCKER | A549 | TRT_CP | dichlorobenzamil, KB-R7943, mibefradil, NNC-55-0396, penfluridol |
| GROWTH_FACTOR_RECEPTOR_INHIBITOR | HCC515 | TRT_CP | GW-441756, danusertib, lestaurtinib |
| LEUCINE_RICH_REPEAT_KINASE_INHIBITOR | A549 | TRT_CP | GW-5074, indirubin, XMD-1150, XMD-885 |

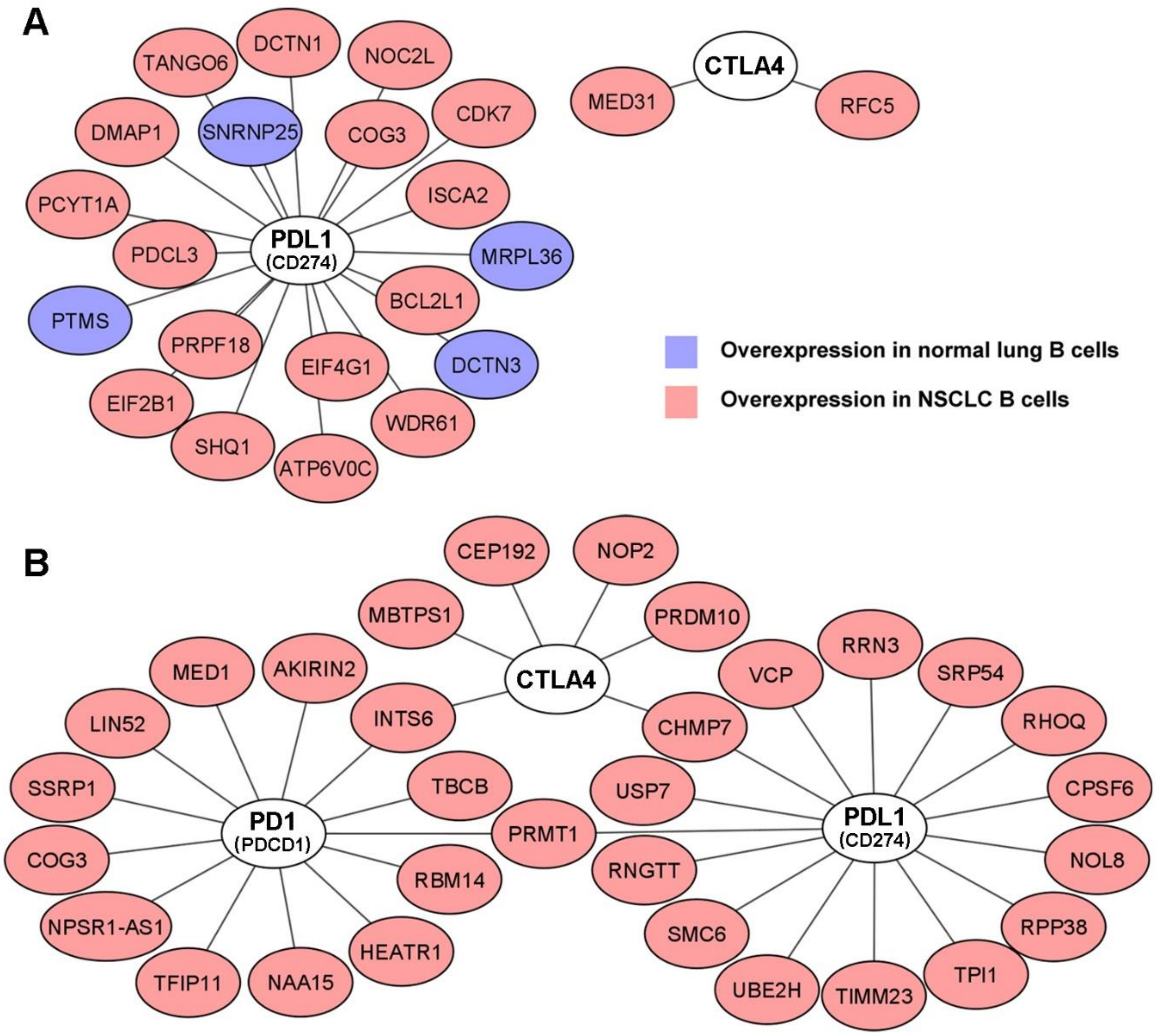


**Supplementary Figure S1. Tumor-specific B-cell proliferation networks involving *PD1 (PDCD1)*, *PDL1 (CD274)*, and *CTLA4* in NSCLC. (A)** Proliferation networks ($p < 0.05$, one-tailed $z$-tests) involving *PD1*, *PDL1*, and *CTLA4* present only in normal lung B cells. **(B)** Proliferation networks ($p < 0.05$, one-tailed $z$-tests) involving *PD1*, *PDL1*, and *CTLA4* present only in NSCLC tumor B cells. Each gene associated with the three immune checkpoint inhibitors was significantly differentially expressed in NSCLC tumor-associated B cells vs. normal lung B cells. Each gene had a significant impact (dependency score < –0.5) on proliferation in CRISPR-Cas9 or RNAi assays in more than 50% of the tested human NSCLC cell lines.

**Supplementary Table S7.** Biological context of edges in Supplementary Figure 1A network (Precision Threshold: 0.6179, Scope Threshold: 0.0278; *p*-value < 0.05; one-tailed *z*-tests).

| **Biological context** | **Precision** | **Scope** |
|---|---|---|
| The up-regulation of PDL1 (CD274) implies the up-regulation of ATP6V0C. | 0.652174 | 0.029948 |
| The up-regulation of PDL1 (CD274) implies the up-regulation of BCL2L1. | 0.652174 | 0.029948 |
| The up-regulation of PDL1 (CD274) implies the up-regulation of CDK7. | 0.652174 | 0.029948 |
| The up-regulation of PDL1 (CD274) implies the up-regulation of COG3. | 0.627907 | 0.027995 |
| The up-regulation of PDL1 (CD274) implies the up-regulation of DCTN1. | 0.627907 | 0.027995 |
| The up-regulation of PDL1 (CD274) implies the up-regulation of DCTN3. | 0.627907 | 0.027995 |
| The up-regulation of PDL1 (CD274) implies the up-regulation of DMAP1. | 0.640449 | 0.028971 |
| The up-regulation of PDL1 (CD274) implies the up-regulation of EIF2B1. | 0.627907 | 0.027995 |
| The up-regulation of PDL1 (CD274) implies the up-regulation of EIF4G1. | 0.648352 | 0.029622 |
| The up-regulation of PDL1 (CD274) implies the up-regulation of ISCA2. | 0.644444 | 0.029297 |
| The up-regulation of PDL1 (CD274) implies the up-regulation of MRPL36. | 0.636364 | 0.028646 |
| The up-regulation of PDL1 (CD274) implies the up-regulation of NOC2L. | 0.636364 | 0.028646 |
| The up-regulation of PDL1 (CD274) implies the up-regulation of PCYT1A. | 0.632184 | 0.02832 |
| The up-regulation of PDL1 (CD274) implies the up-regulation of PDCL3. | 0.648352 | 0.029622 |
| The up-regulation of PDL1 (CD274) implies the up-regulation of PRPF18. | 0.632184 | 0.02832 |
| The up-regulation of PDL1 (CD274) implies the up-regulation of PTMS. | 0.636364 | 0.028646 |
| The up-regulation of PDL1 (CD274) implies the up-regulation of SHQ1. | 0.640449 | 0.028971 |
| The up-regulation of PDL1 (CD274) implies the up-regulation of SNRNP25. | 0.640449 | 0.028971 |
| The up-regulation of PDL1 (CD274) implies the up-regulation of TANGO6. | 0.632184 | 0.02832 |
| The up-regulation of PDL1 (CD274) implies the up-regulation of WDR61. | 0.636364 | 0.028646 |
| The up-regulation of CTLA4 implies the up-regulation of MED31. | 0.632184 | 0.02832 |
| The up-regulation of CTLA4 implies the up-regulation of RFC5. | 0.632184 | 0.02832 |

**Supplementary Table S8.** Biological context of edges in Supplementary Figure 1B network (Precision Threshold: 0.6179, Scope Threshold: 0.0278; *p*-value < 0.05; one-tailed *z*-tests).

| **Biological context** | **Precision** | **Scope** |
|---|---|---|
| The up-regulation of PDL1 (CD274) implies the up-regulation of CHMP7. | 0.725714 | 0.037977 |
| The up-regulation of PDL1 (CD274) implies the up-regulation of CPSF6. | 0.695238 | 0.03418 |
| The up-regulation of PDL1 (CD274) implies the up-regulation of NOL8. | 0.709091 | 0.035807 |
| The up-regulation of PDL1 (CD274) implies the up-regulation of PRMT1. | 0.704615 | 0.035265 |
| The up-regulation of PDL1 (CD274) implies the up-regulation of RHOQ. | 0.657143 | 0.030382 |
| The up-regulation of PDL1 (CD274) implies the up-regulation of RNGTT. | 1 | 0.029839 |
| The up-regulation of PDL1 (CD274) implies the up-regulation of RPP38. | 0.704615 | 0.035265 |
| The up-regulation of PDL1 (CD274) implies the up-regulation of RRN3. | 0.685246 | 0.033095 |
| The up-regulation of PDL1 (CD274) implies the up-regulation of SMC6. | 0.690323 | 0.033637 |
| The up-regulation of PDL1 (CD274) implies the up-regulation of SRP54. | 1 | 0.028212 |
| The up-regulation of PDL1 (CD274) implies the up-regulation of TIMM23. | 0.690323 | 0.033637 |
| The up-regulation of PDL1 (CD274) implies the up-regulation of TPI1. | 0.650909 | 0.029839 |
| The up-regulation of PDL1 (CD274) implies the up-regulation of UBE2H. | 1 | 0.032552 |
| The up-regulation of PDL1 (CD274) implies the up-regulation of USP7. | 0.668966 | 0.031467 |
| The up-regulation of PDL1 (CD274) implies the up-regulation of VCP. | 0.657143 | 0.030382 |

| | | |
|---|---|---|
| The up-regulation of CTLA4 implies the up-regulation of CEP192. | 0.661972 | 0.030816 |
| The up-regulation of CTLA4 implies the up-regulation of CHMP7. | 0.657143 | 0.030382 |
| The up-regulation of CTLA4 implies the up-regulation of INTS6. | 0.630769 | 0.028212 |
| The up-regulation of CTLA4 implies the up-regulation of MBTPS1. | 0.641791 | 0.02908 |
| The up-regulation of CTLA4 implies the up-regulation of NOP2. | 0.671233 | 0.031684 |
| The up-regulation of CTLA4 implies the up-regulation of PRDM10. | 0.688312 | 0.03342 |
| The up-regulation of PD1 (PDCD1) implies the up-regulation of AKIRIN2. | 0.661972 | 0.030816 |
| The up-regulation of PD1 (PDCD1) implies the up-regulation of COG3. | 0.671233 | 0.031684 |
| The up-regulation of PD1 (PDCD1) implies the up-regulation of HEATR1. | 0.7 | 0.034722 |
| The up-regulation of PD1 (PDCD1) implies the up-regulation of INTS6. | 0.630769 | 0.028212 |
| The up-regulation of PD1 (PDCD1) implies the up-regulation of LIN52. | 1 | 0.028212 |
| The up-regulation of PD1 (PDCD1) implies the up-regulation of MED1. | 0.652174 | 0.029948 |
| The up-regulation of PD1 (PDCD1) implies the up-regulation of NAA15. | 0.630769 | 0.028212 |
| The up-regulation of PD1 (PDCD1) implies the up-regulation of NPSR1-AS1. | 0.666667 | 0.03125 |
| The up-regulation of PD1 (PDCD1) implies the up-regulation of PRMT1. | 0.630769 | 0.028212 |
| The up-regulation of PD1 (PDCD1) implies the up-regulation of RBM14. | 1 | 0.032552 |
| The up-regulation of PD1 (PDCD1) implies the up-regulation of SSRP1. | 0.7 | 0.034722 |
| The up-regulation of PD1 (PDCD1) implies the up-regulation of TBCB. | 0.652174 | 0.029948 |
| The up-regulation of PD1 (PDCD1) implies the up-regulation of TFIP11. | 0.7 | 0.034722 |
